# The paraspecific neutralisation of snake venom induced coagulopathy by antivenoms

**DOI:** 10.1038/s42003-018-0039-1

**Published:** 2018-04-19

**Authors:** Stuart Ainsworth, Julien Slagboom, Nessrin Alomran, Davinia Pla, Yasir Alhamdi, Sarah I. King, Fiona M. S. Bolton, José María Gutiérrez, Freek J. Vonk, Cheng-Hock Toh, Juan J. Calvete, Jeroen Kool, Robert A. Harrison, Nicholas R. Casewell

**Affiliations:** 1 Alistair Reid Venom Research Unit, Parasitology Department, Liverpool School of Tropical Medicine, Pembroke Place, Liverpool, L3 5QA UK; 2 Division of BioAnalytical Chemistry, Department of Chemistry and Pharmaceutical Sciences, Faculty of Sciences, Vrije Universiteit Amsterdam, Amsterdam, 1081 LA The Netherlands; 3 Laboratorio de Venómica Estructural y Funcional, Instituto de Biomedicina de Valencia, CSIC, Valencia, 46010 Spain; 4 Institute of Infection and Global Health, University of Liverpool, Liverpool, L69 7BE UK; 5 Instituto Clodomiro Picado, Facultad de Microbiología, Universidad de Costa Rica, San José, 11501-2060 Costa Rica; 6 Naturalis Biodiversity Center, 2333 CR Leiden, The Netherlands; 7 Roald Dahl Haemostasis and Thrombosis Centre, Royal Liverpool University Hospital, Liverpool, L7 8XP UK; 8 Research Centre for Drugs and Diagnostics, Liverpool School of Tropical Medicine, Pembroke Place, Liverpool, L3 5QA UK

## Abstract

Snake envenoming causes several potentially lethal pathologies. The specific pathology is dictated by the toxin composition of venom, which varies by species, geography and ontogeny. This variation severely restricts the paraspecific efficacy of antivenoms used to treat snakebite victims. With a view to devising pathology-specific snakebite treatments, we assessed the procoagulant activity of 57 snake venoms and investigated the efficacy of various antivenoms. We find that procoagulant venoms act differentially on key steps of the coagulation cascade, and that certain monospecific antivenoms work in a previously unrecognised paraspecific manner to neutralise this activity, despite conventional assumptions of congener-restricted efficacy. Moreover, we demonstrate that the metal chelator EDTA is also capable of neutralising venom-induced lethality in vivo. This study illustrates the exciting potential of developing new, broad-spectrum, toxin-targeting antivenoms capable of treating key snakebite pathologies, and advocates a thorough re-examination of enzyme inhibiting compounds as alternative therapies for treating snakebite victims.

## Abstract (web summary)

Stuart Ainsworth and colleagues characterized the coagulopathic activity of snake venom and demonstrated that certain monospecific antivenoms can neutralize procoagulant venoms from more than one species. This study suggests a possibility of developing broad-spectrum, toxin-targeting antivenoms to treat snakebite victims.

## Introduction

Venomous snakes possess some of the most potent biochemical weapons found in the animal kingdom^1^. Their venom consists of mixtures of bioactive proteinacious components (circa. 50–200 per species) that vary inter- and intra-specifically and function to immobilise and/or kill prey^1–4^. Snakes can also deploy their venom defensively, and such bites result in 100,000 deaths each year, with 3–5 times that number of people suffering from long-term morbidity. Consequently, snakebite is one of the world’s most lethal neglected tropical diseases^5–7^.

The only specific therapies currently available for the treatment of snakebite are antivenoms, which consist of polyclonal immunoglobulins purified from sera/plasma of horses or sheep immunised with snake venom(s). Because of inter-specific venom variation, antivenoms are fundamentally limited in their efficacy to those species whose venom was used for immunisation or, in some cases, relatively few closely related species that possess highly similar venom components^8–10^. Consequently, many different antivenom therapies exist across and within different continents, each with varying efficacies to different snake species^11,12^.

Snake venoms cause a variety of different effects in human victims, including neurotoxic, haemotoxic, cytotoxic, myotoxic and/or coagulopathic pathologies^7,13^. Of these, venom-induced consumption coagulopathy, caused by procoagulant snake venoms, is said to be one of the most common medically important snakebite pathologies^14^. This haemostatic alteration is characterised clinically by the depletion of fibrinogen, and caused by venom toxins continually activating and consuming various clotting factors in the coagulation cascade^14,15^. Such severe coagulopathy makes snakebite victims particularly vulnerable to suffering life-threatening haemorrhage^14^.

To improve our understanding of the spectrum of snakes causing venom-induced consumption coagulopathy, their mechanisms of action and to expand therapeutic options, here we characterise the procoagulant activity of venom sourced from a wide range of diverse snake species and investigate the extent to which antivenom and the metal chelator EDTA (ethylenediaminetetraacetic acid) are capable of neutralising these effects across species (paraspecificity). Our results provide support for the development of new “pathology-specific” snakebite treatments capable of neutralising key venom toxicities irrespective of the snake species responsible for envenoming.

## Results

### Venom activity on plasma, Factor X, prothrombin and fibrinogen

**Fig. 1:**
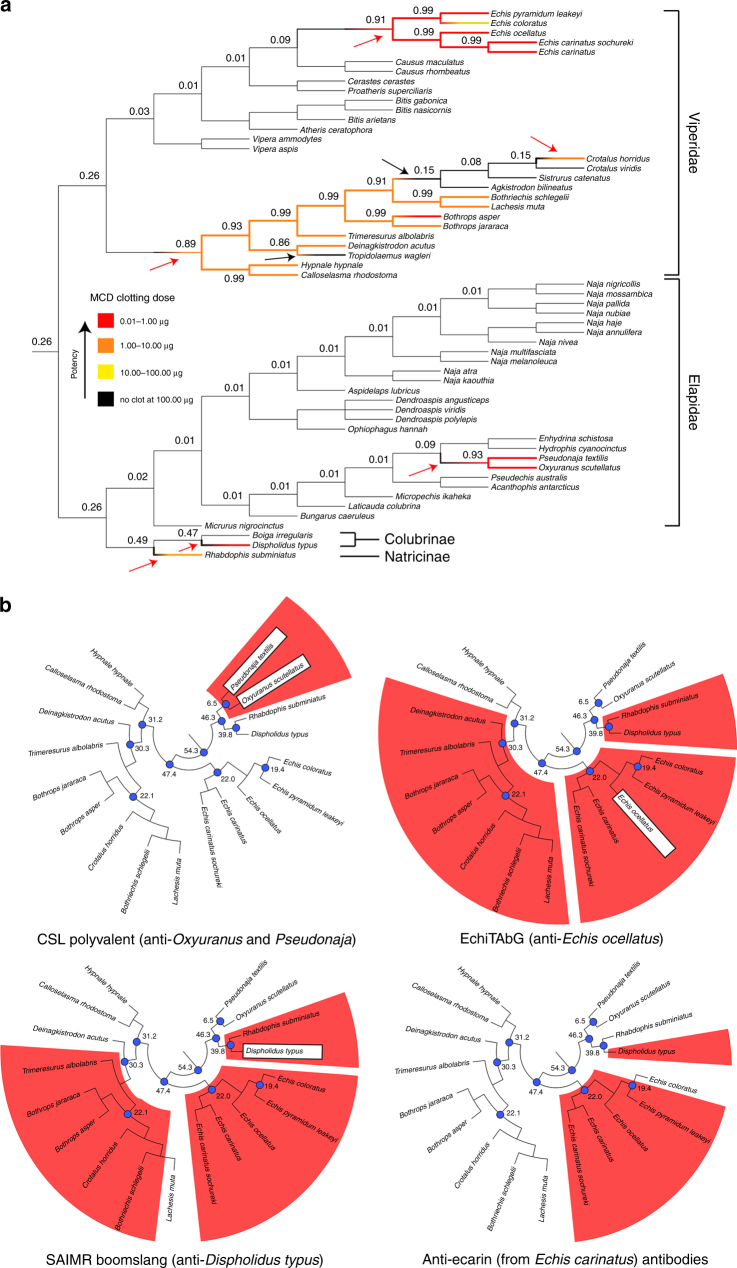
Convergent evolution of procoagulant venom activity and in vitro neutralisation by antivenoms. **a** The convergent evolution of procoagulant venom function and the potency of the snake venoms used in this study overlaid onto a species phylogeny (cladogram). Procoagulant venom activity has evolved independently on at least six occasions (red arrows) in the advanced snakes. Black arrows indicate loss events. Colouring of branches indicates the procoagulant potency as defined in the key. Numbers at key nodes represent the proportional likelihoods of procoagulant venom function being the ancestral state at that node. **b** The neutralisation of procoagulant venom activity in the plasma assay by various antivenoms overlaid onto species trees pruned to include only those venoms found to be procoagulant. Red shading highlights neutralisation of coagulation. The species used to raise the various antivenom antibodies are highlighted in white boxes. Divergence times (millions of years) are indicated at key blue coloured nodes on the tree: 54.3, base of the advanced snake radiation; 47.4, base of viper radiation; 31.2, 30.3, 22.1, 22.0 and 19.4, key internal nodes within vipers; 46.3, split of elapids from colubrids and natricines; 39.8, split of colubrids from natricines; 6.5, split of *Psuedonaja* and *Oxyuranus*. For both sets of trees, the species relationships and divergence times were reconstructed from previous studies^23, 55–58^. See also Supplementary Tables 1–3

We first screened the procoagulant bioactivity of 57 venoms sourced from a variety of phylogenetically and geographically diverse snake species (Supplementary Table 1) in a minimum coagulant dose plasma (MCD-P) assay^16^. Eighteen of the 57 venoms exhibited procoagulant activities at the maximal dose (100 μg), and without the addition of cofactors, such as calcium. These procoagulant venoms included representatives from all four snake families/subfamilies tested and they exhibited considerable variation in potency (Fig. 1a, Supplementary Table 2). Reconstructing the evolutionary history of procoagulant venom activity demonstrated that this functional phenotype has evolved convergently; originating on at least six independent occasions in snakes, three times in vipers (including at least two losses), once in elapids, once in colubrids and once in natricines (Fig. 1a).

Various snake venoms have previously been described to contain toxins that act on components of the blood clotting cascade, including Factors V, VII, X, II (prothrombin) and I (fibrinogen)^17,18^. The latter three are well-known targets for procoagulant venom toxins and, consequently, their properties have been exploited for use as diagnostic and therapeutic tools in human medicine^17,18^. To determine the specific action of each of the 18 procoagulant venoms on Factor X, prothrombin and fibrinogen, we compared their activity in (i) chromogenic enzymatic, (ii) degradation gel electrophoretic and (iii) factor-deficient plasma coagulation assays.

**Fig. 2:**
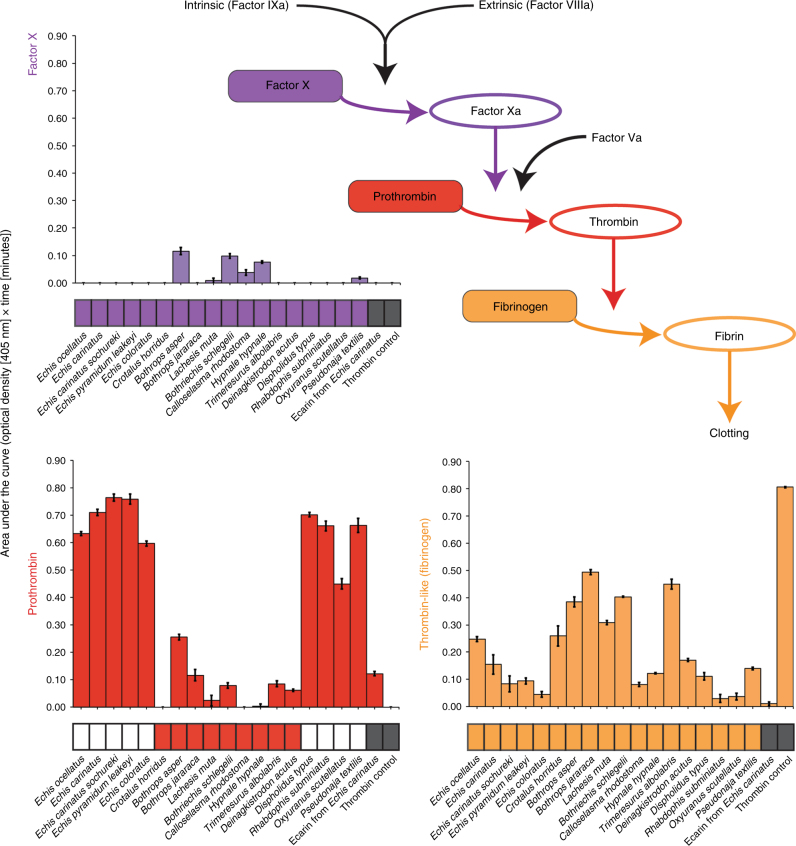
The bioactivity of procoagulant snake venoms on key components of the coagulation cascade. The coagulation cascade schematic highlights the end of this pathway and the coagulation factors tested in our chromogenic enzyme assay. Bar charts display the functional activity of each procoagulant venom, the snake venom metalloproteinase toxin ecarin, and a thrombin control against Factor X (purple), prothrombin (red) and fibrinogen (orange). Bars represent areas under the curve of optical density (405 nm) plotted against time. Error bars represent SEM of triplicate measurements. Below each bar chart are blocks that indicate whether each venom induced clot formation in: (i) plasma deficient in Factor X (purple), (ii) plasma deficient in prothrombin (red) and normal plasma (orange) at the MCD-P dose. Coloured blocks indicate clot formation and white blocks indicate no clot formation at ten times the MCD-P dose. Grey blocks (for ecarin and thrombin) indicate not tested. See Supplementary Figs. 7 and 8 for the plotted data used for area under the curve calculations

None of the venoms tested exhibited a strong effect on Factor X (Fig. 2, Supplementary Fig. 1), and all induced a clot in Factor X-deficient plasma at the same dose as normal plasma (Fig. 2, Supplementary Table 2). In contrast, degradation assay results demonstrated that all 18 venoms enzymatically cleave prothrombin and, for some, resulted in cleavage-products with comparable masses to meizothrombin and/or thrombin, consistent with activation (Supplementary Fig. 2). However, the chromogenic enzyme assay demonstrated that only nine venoms (five *Echis* spp.*, Dispholidus typus*, *Oxyuranus scutellatus, Pseudonaja textilis* and *Rhabdophis subminiatus*) were potent prothrombin activators (Fig. 2), and all were incapable of coagulating prothrombin-deficient plasma, even when using venom doses tenfold higher than that required to coagulate normal plasma (Supplementary Table 2).

We next measured the action of each venom on fibrinogen, which is typically cleaved by thrombin to generate fibrin and cross-linked stable clots. All 18 procoagulant venoms exhibited some degree of thrombin-like enzyme activity and several venoms with low prothrombin-activating potency had the highest thrombin-like enzyme activity (e.g. *Lachesis muta*, *Trimeresurus albolabris*) (Fig. 2). In contrast to venom thrombin-like enzymes promoting fibrin clot formation, other venom enzymes degrade fibrinogen chains resulting in non-functional clots. Fibrinogen-degradation profiles of the 18 venoms revealed variant fibrinogenolytic activity—while the majority cleaved the α chain of fibrinogen, some also cleaved the β chain (Supplementary Fig. 3).

In combination, these results illustrate that some snakes have evolved multiple venom proteins with distinct specificities that simultaneously target several key coagulation molecules to cause continual activation, and hence consumption, of key components of the clotting cascade. An example is *Bothrops asper* venom, which acts moderately on Factor X, prothrombin and fibrinogen^19,20^ (Fig. 2). Other venoms only target specific components; thus, *R. subminiatus* venom is a potent prothrombin-activator but exhibits little/no activity on Factor X or fibrinogen (Fig. 2). We next wished to assess the extent to which antivenom, the only specific therapy for treating snakebite, might exhibit paraspecific neutralising capabilities against the toxins causing procoagulant effects. If similar toxins in different snake species are responsible for coagulopathy, we might expect some degree of cross-reactivity and cross-neutralisation, whereas distinct, taxon-specific, toxins would likely result in preclinical antivenom failure, as typically reported^8–10^.

### Antivenom cross-reactivity and neutralisation of coagulation

We assessed venom neutralisation in the plasma assay using three antivenoms designed to neutralise the lethal effects of highly procoagulant venoms from three different snake families, specifically: “EchiTAbG” (anti-*Echis ocellatus*, family Viperidae), “SAIMR boomslang” (anti-*D. typus*, family Colubridae) and “CSL polyvalent” (anti-Australian Elapidae snakes, including *O. scutellatus* and *P. textilis*). In addition, to assess the paraspecific immunological cross-reactivity of each antivenom, we performed immunoblotting experiments with the various antivenoms and each of the procoagulant venoms.

**Fig. 3:**
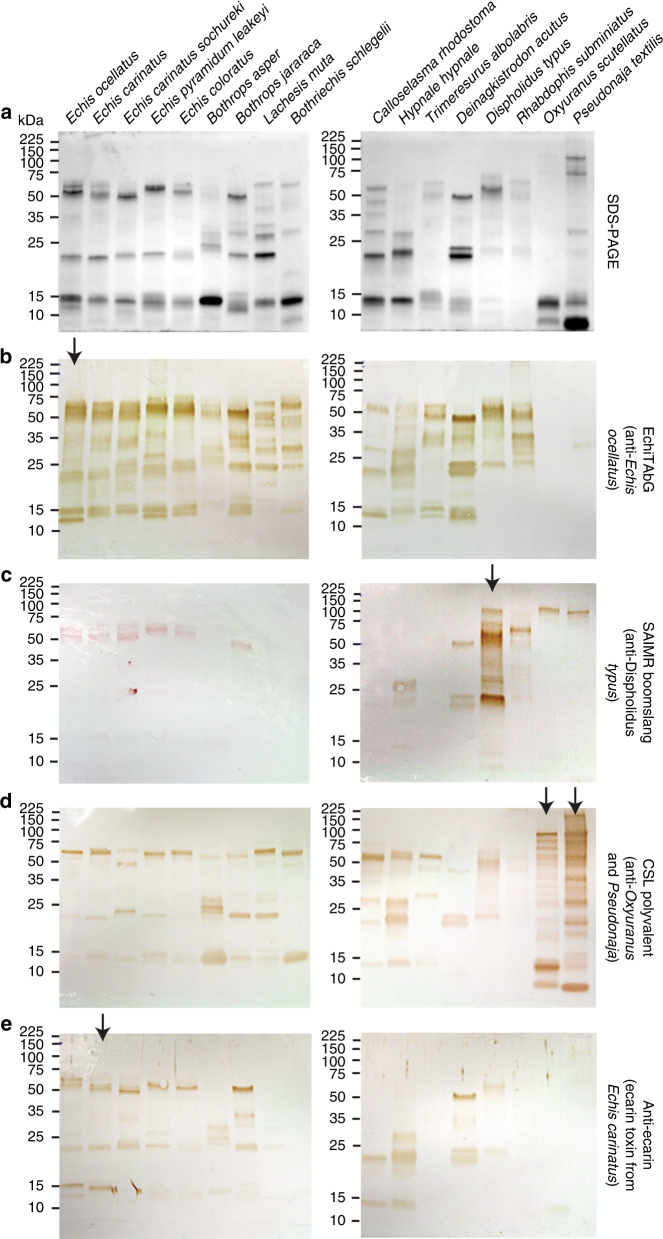
The immunological cross-reactivity of antivenoms and toxin-specific antibodies with a diverse range of procoagulant snake venoms. **a** Reduced SDS-PAGE gel electrophoretic profiles of procoagulant snake venoms showing extensive venom variation and **b−e** their cross-reactivity with various antivenoms via western blotting. Arrows represent the species whose venom (or, in the case of ecarin, toxin) was used to generate the antibodies

The CSL polyvalent antivenom was highly effective at neutralising the in vitro procoagulant effects of the two elapid snakes tested (*O. scutellatus* and *P. textilis*), but ineffective against all other venoms (Fig. 1b, Supplementary Table 3). Immunoblotting the CSL antivenom against each venom demonstrated low levels of toxin cross-reactivity, except to those two elapid venoms used for immunisation (Fig. 3). These results are consistent with our expectations of limited antivenom paraspecificity as a consequence of venom variation^21^.

The anti-*E. ocellatus* antivenom, EchiTAbG, prevented venom-induced coagulation by all five *Echis*-genus saw-scaled vipers, which was unsurprising given prior reports of intra-generic cross-reactivity^3,8,10^ (although see ref. ^22^). Notably, EchiTAbG also neutralised the procoagulant venom effects of seven of the remaining nine viper species, the colubrid snake *D. typus* and the natricine *R. subminiatus*, despite these latter two species having diverged from vipers over 54 million years ago^23^ (Fig. 1b, Supplementary Table 3). These paraspecific neutralising results are particularly surprising since the efficacy of monospecific antivenom (those made against a single snake species) is typically restricted to congeners. The assay conditions of in vitro pre-incubating venoms and antivenoms prior to assessment of neutralisation, and the unusually extensive venom cross-reactivity of the monospecific EchiTAbG antivenom (Fig. 3), likely underpins the paraspecific neutralising capability of this antivenom.

The SAIMR boomslang antivenom also provided a surprisingly high degree of paraspecific neutralisation by preventing coagulation caused by venoms of 10/14 vipers and *R. subminiatus* and *D. typus* (Supplementary Table 3). The immunoblot illustrated that cross-specific immunoreactivity of this antivenom was predominately restricted to venom proteins 50–65 kDa in size (Fig. 3). These likely correspond to zinc-dependent snake venom metalloproteinase (SVMP) toxins, several of which are known to encode isoforms possessing procoagulant properties^17,18^.

To explore this further, we used antibodies previously raised^24^ by immunising rabbits with the prothrombin-activating ~56 kDa SVMP ecarin (isolated from *Echis carinatus* venom^25^) in the same assays. First, we confirmed that ecarin is a procoagulant venom toxin by demonstrating that it activates and cleaves prothrombin (Fig. 2, Supplementary Figs. 1–3). The anti-ecarin antibody cross-reacted with viper venom proteins of diverse masses, all presumably SVMPs, and notably, to some of the same masses (~50–65 kDa) as those bound by the SAIMR boomslang antivenom (Fig. 3). Notably, for such a protein-specific antibody, we found that the anti-ecarin antibody neutralised the procoagulant venom activity of venom from multiple *Echis* spp. and *D. typus* in the plasma assay (Fig. 1b, Supplementary Table 3).

### In vitro neutralisation of saw-scaled viper and boomslang venom

We next investigated how antibodies raised separately against venom from the saw-scaled viper (*E. ocellatus*), the colubrid boomslang (*D. typus*) and the SVMP toxin ecarin are capable of reciprocally neutralising the procoagulant function of venom from these two highly divergent (split >54 million years ago) snake species. The results of the plasma assay were somewhat unexpected because the toxin composition of snake venoms are known to vary extensively at every taxonomic level due to a variety of processes^2–4,26–28^, and these are well known to undermine the paraspecific efficacy of antivenom^8,9,29^.

**Fig. 4:**
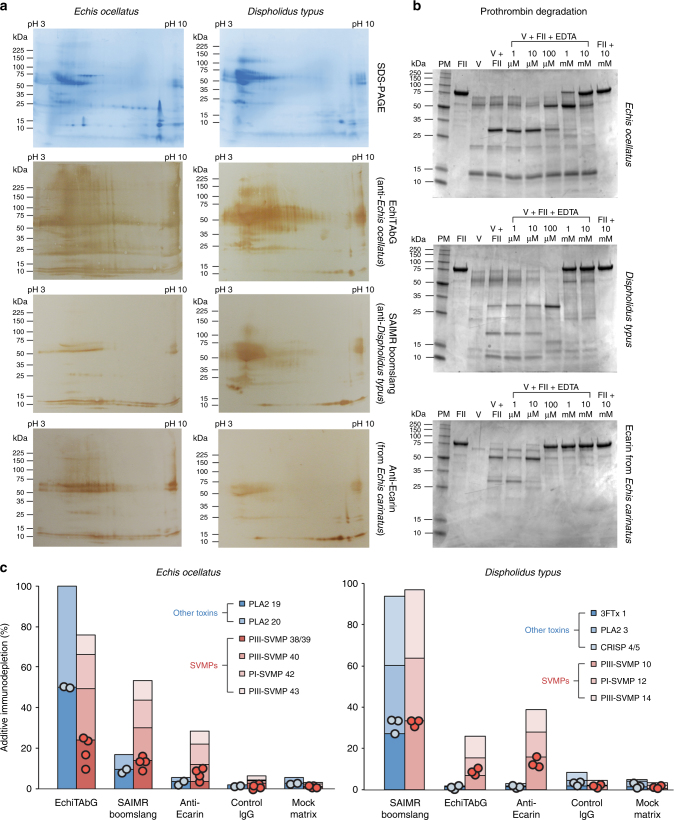
The immunological cross-reactivity and neutralisation of *Echis ocellatus* and *Dispholidus typus* venom by antivenom is mediated by interactions with snake venom metalloproteinases. **a** Two-dimensional SDS-PAGE gel electrophoresis profiles of venom and their cross-reactivity with species-specific and paraspecific antibodies detected by western blotting. **b** Inhibition of venom and toxin-induced degradation of prothrombin by the metalloproteinase inhibitor EDTA. For both venoms and ecarin 1 mM of EDTA inhibits the complete degradation of prothrombin. Key: PM protein marker, FII prothrombin, V venom (ecarin for the ecarin experiment); numbers indicate the molarity of EDTA used in increasing concentrations from left to right. **c** The percentage immunodepletion of venom toxins bound by the antivenoms used in the antivenomic experiments. Comparisons of the immunodepletion of SVMPs with non-SVMP toxins demonstrate that the heterologous antibody preparations (e.g. SAIMR boomslang vs. *Echis ocellatus*; EchiTAbG and anti-ecarin vs. *Dispholidus typus*) predominately immunodeplete (bind to) SVMPs. Circles represent data points of the immunodepletion percentage of the individual toxins within each toxin group analysed (SVMPs or other toxins), and bars are additive for those data points found within each group. See also Supplementary Figs. 4 and 5

Envenoming by both *E. ocellatus* and *D. typus* cause similar haemorrhage and consumption coagulopathy syndromes in human victims^14,30,31^, and we previously demonstrated that both these venoms are dominated by SVMP toxins^3,32,33^, some of which are prothrombin activators^25,34^. We confirmed this here by incubating both venoms with different concentrations of EDTA, which chelates zinc (and other metals), to inhibit the bioactivity of zinc-dependent SVMPs, before repeating our prothrombin degradation gels. We demonstrate that 1 mM of EDTA was sufficient to begin preventing degradation of prothrombin by each venom and also by the calcium-independent prothrombin-activating SVMP ecarin (Fig. 4). As both *E. ocellatus* and *D. typus* venoms have little/no activity on Factor X and low/moderate activity on fibrinogen, but potently activate prothrombin (Fig. 2, Supplementary Figs. 1–3), these results strongly suggest that SVMPs are predominately responsible for the procoagulant activities caused by these two venoms.

Despite their divergence from one another over 50 million years ago, 2D gel electrophoretic venom profiles demonstrated many similarities between the venoms of *E. ocellatus* and *D. typus* (Fig. 4). Probing these venom profiles with the two species-specific antivenoms (EchiTAbG and SAIMR boomslang) revealed extensive immunological recognition, including paraspecifically (Fig. 4). Moreover, the SVMP-specific anti-ecarin antibody demonstrated that both venoms are dominated by SVMP toxins of varying molecular weights, and provided additional evidence^35^ of the extensive cross-specific reactivity of antibodies raised to single SVMP toxins (Fig. 4).

**Fig. 5:**
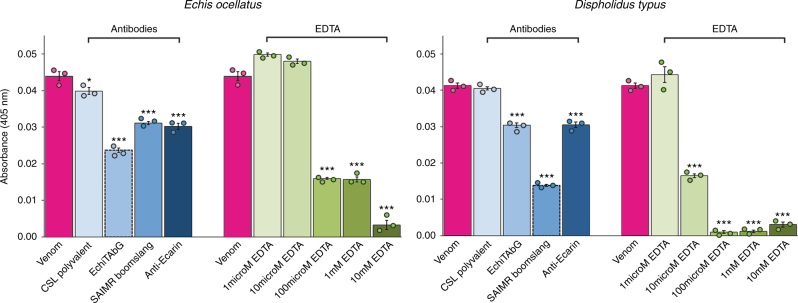
The neutralisation of venom-induced prothrombin activation by homologous and heterologous antivenoms and EDTA. The effect of antibodies (150 μg) and EDTA (various molarities from 1 μM to 10 mM) on venom-induced prothrombin activation measured by chromogenic assay. Bars represent end-point absorbances (405 nm). Error bars represent SEM of triplicate measurements. Circles represent individual data points. Asterisks indicate significant reductions in activity compared to venom-only measurements: ***P < 0.001, **P < 0.01, *P < 0.05; one-way ANOVA with Tukey’s HSD post hoc test

We further explored these venom protein−antibody interactions by using an “antivenomics” approach^36^ to identify the venom proteins that bind to each antivenom. The three antibody preparations were separately immobilised on sepharose columns, venom added, and bound and unbound venom toxins identified by comparing the resulting reverse-phase liquid chromatographic profiles with whole venom previously characterised by mass spectrometry-based venomic analyses^32,33^. As anticipated, the homologous venom−antivenom combinations (e.g. *E. ocellatus*/EchiTAbG and *D. typus*/SAIMR boomslang) effectively bind the vast majority of venom proteins, whereas the heterologous combinations recognise and bind to SVMP toxins to a much greater extent than to other toxin types (Fig. 4, Supplementary Figs. 4 and 5). This suggests that it is the SVMP-specific antibodies in the EchiTAbG and SAIMR boomslang antivenoms (and the anti-ecarin antibody) that neutralise the procoagulant venom effects of *E. ocellatus* and *D. typus* (Figs. 1b, 4, Supplementary Table 3). Supporting this assertion is evidence that each antivenom significantly reduces the prothrombin activating activity of *E. ocellatus* and *D. typus* venom in the chromogenic assay (all *P* < 0.001 compared to venom-only and CSL antivenom controls) (Fig. 5).

### In vivo neutralisation of saw-scaled viper and boomslang venom

**Fig. 6:**
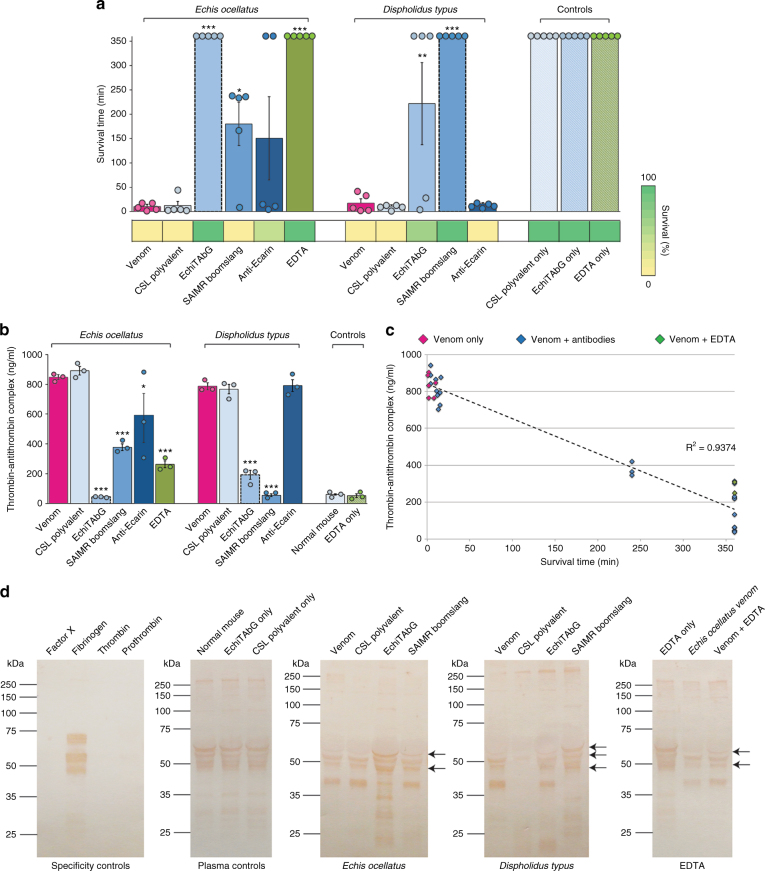
In vivo neutralisation of *Echis ocellatus* and *Dispholidus typus* venoms by homologous and heterologous antivenoms and EDTA. **a** The survival and duration of survival of mice treated with venom and antibodies or EDTA. Bars represent mean survival times in minutes (circles represent individual data points and error bars indicate SEM; *n* = 5) and coloured blocks below the chart indicate percentage survival (*n* = 5). Homologous antivenom−venom combinations are indicated by dashed lines around bars. Asterisks indicate significant increases in survival time compared to venom-only measurements: ***P < 0.001, **P < 0.01, *P < 0.05; one-way ANOVA with Tukey’s HSD post hoc test. **b** The concentration of thrombin−antithrombin complexes (TAT) in plasma from mice treated with venom and antibodies or EDTA. Circles represent individual data points and error bars indicate SEM of triplicate measurements. Homologous antivenom−venom combinations are indicated by dashed lines around bars. Asterisks indicate significant reductions in TAT concentrations compared to venom-only measurements: ***P < 0.001, **P < 0.01, *P < 0.05; one-way ANOVA with Tukey’s HSD post hoc test. **c** TAT levels show a strong inverse relationship with survival times (*R*^2^ = 0.9374). Note that results from controls (antivenom only, EDTA only and normal mouse pre- and post-study) are not included in this analysis as these animals received no venom. Inclusion of these data points results in a drop in *R*^2^ to 0.5203, demonstrating that the correlation observed is a result of treatment intervention and not time (i.e. factor regeneration over time). **d** Immunoblotting plasma from mice treated with venom and antibodies or EDTA demonstrates variation in circulating fibrinogen. Arrows highlight key variable bands (either in terms of presence or intensity) visualised with anti-fibrinogen antibodies in each panel. Mouse plasma was pooled from all experimental animals in each treatment group (*n* = 5). See Supplementary Fig. 6 for details of the same immunoblotting experiments using anti-prothrombin antibodies

Given these promising in vitro levels of antivenom cross-reactivity and neutralisation, we next tested whether the heterologous venom−antivenom combinations were capable of neutralising venom lethality in vivo, while acknowledging the potential for toxicity conferred by other non-coagulopathic venom toxins. We challenged groups of five mice intravenously with 2.5 × the 50% lethal dose (LD_50_) of each venom (*E. ocellatus*—17.85 μg [95% confidence intervals 12.46–28.53]; *D. typus*—22.29 μg [9.12–47.96]) pre-incubated with 7.5 mg (375 μg/g) of each antivenom. Venom-only controls succumbed to the lethal venom effects within 20 min, whereas antivenom-only controls survived until the experiment end (360 min) (Fig. 6). As anticipated, the homologous venom and antivenom combinations (*E. ocellatus*/EchiTAbG and *D. typus*/SAIMR boomslang) also resulted in complete survival, while the CSL polyvalent antivenom, used as a non-specific antivenom control, provided no protection against either venom (Fig. 6).

Notably, the heterologous antivenoms provided some in vivo protection against the lethal effects of each venom, supporting the results of our earlier in vitro coagulation-specific assays. Thus, while mice subjected to the *E. ocellatus* venom and SAIMR boomslang antivenom combination ultimately succumbed to the lethal venom effects during the 6 h experimental period, we found a significant delay in the onset of lethality, from 10.6 min (venom-only) to 180.0 min (*P* = 0.039) (Fig. 6). Similarly, mice treated with the *D. typus* venom and EchiTAbG antivenom combination also exhibited significantly prolonged survival (17.4 min, venom-only; 222.0 min, venom and EchiTAbG; *P* = 0.002), and three of the five experimental animals survived the duration of the experiment (Fig. 6). The anti-ecarin antibodies failed to protect mice envenomed with *D. typus* venom, but showed some neutralising capability against *E. ocellatus* venom, with two animals surviving the duration of the experiment (Fig. 6), although no significant difference in mean survival times were observed (*P* *=* 0.134). It is, however, notable that antibodies generated against a single venom toxin were capable of offering some protection against the lethal effects of snake venom in vivo, and these results are therefore encouraging for the future design of highly specific, monoclonal antibody-based therapies for snakebite. Furthermore, in agreement with recent microarray studies that mapped toxin epitope and antivenom interactions^37,38^, these results provide additional, in vivo evidence that mixtures of different recombinant monoclonal or oligoclonal antibodies, targeting different toxin types/isoforms, will almost certainly be required to effect cure^39^.

We next used plasma from euthanised experimental animals to assess markers of coagulopathy. Specifically, we quantified the concentration of thrombin−antithrombin complexes (TAT), which is a sensitive marker for in vivo thrombin generation^40^, and we probed plasma profiles (Supplementary Fig. 6) with anti-prothrombin and anti-fibrinogen antibodies in immunoblotting experiments. Venom-only controls resulted in profiles of extensively degraded fibrinogen and prothrombin, and extremely high levels of TAT (>750 ng/ml), compared with normal mouse and antivenom-only controls (~60 ng/ml) (Fig. 6, Supplementary Fig. 6). Animals receiving homologous venom/antivenom combinations exhibited significantly lower TAT levels (45–55 ng/ml; *P* < 0.001 for both) and recovery of intact fibrinogen and prothrombin. Moreover, administration of the heterologous SAIMR boomslang and anti-ecarin antivenoms against *E. ocellatus* venom also resulted in significant reductions in TAT levels (*P* < 0.001 and *P* = 0.014 respectively), while EchiTAbG antivenom significantly reduced TAT levels when compared to mice receiving *D. typus* venom (*P* < 0.001) (Fig. 6). TAT levels therefore exhibit a strong inverse relationship with the survival times (*R*^2^ = 0.9374) of experimental mice (Fig. 6). While none of the non-homologous venom−antivenom combinations recovered prothrombin profiles comparable to normal mouse controls (Supplementary Fig. 6), we observed some recovery of fibrinogen in mice recipient to *E. ocellatus* venom/SAIMR boomslang and *D. typus* venom/EchiTAbG combinations, and specifically a protein that corresponds with the mass of the β chain of fibrinogen (~54 kDa) (Fig. 6). In combination, these experiments provide evidence that the heterologous antivenom antibodies likely prolong survival via the neutralisation of coagulopathic toxins, thereby preventing the complete depletion of fibrinogen and retaining some degree of coagulation.

### Neutralisation of saw-scaled viper and boomslang venom by EDTA

Following our demonstration that EDTA effectively prevented complete degradation of prothrombin by *E. ocellatus* and *D. typus* venoms (Fig. 4), we repeated the prothrombin chromogenic assay with each venom preincubated with various concentrations of EDTA instead of antivenom. We found that 100 μM of EDTA significantly reduced the procoagulant activity of each venom when compared to results using the two species-appropriate antivenoms (both *P* < 0.001) (Fig. 5).

Although EDTA has long been used as an experimental tool to inhibit zinc-dependent SVMPs in vitro, along with a handful of reports of EDTA inhibiting the intradermal haemorrhagic and necrotic activity of *E. ocellatus* and *B. asper* venoms^41–43^, its potential utility at preventing lethality has never been demonstrated. Encouraged by our in vitro results, we tested whether EDTA was capable of preventing in vivo lethality caused by *E. ocellatus* venom. This venom was selected because it represents a more severe model of murine envenoming than *D. typus*, its venom composition is more diverse (including containing many more non-SVMP toxins)^3,32,33^, and this genus arguably causes more snakebite deaths each year than any other group of snakes^30^.

As described above for the antibody study, we pre-incubated EDTA (100 μg (1.71 mM); 5 μg/g) with 2.5 × LD_50_ doses of *E. ocellatus* venom before intravenously injecting five mice, alongside an EDTA-only control group. All animals in the control group survived until the experiment end (360 min) and exhibited normal TAT levels and prothrombin and fibrinogen profiles (Fig. 6, Supplementary Fig. 6), confirming that this dose of EDTA is non-toxic in mice^44^. All mice in the experimental group receiving the venom−EDTA combination also survived until the end of the experiment, demonstrating, for the first time to our knowledge, that EDTA effectively protects against the lethal effects of *E. ocellatus* venom (mean survival, 360 min; venom-only control, 10.6 min; *P* < 0.001) (Fig. 6). Furthermore, plasma TAT levels in venom/EDTA-treated mice were significantly lower than those from venom-only controls (*P* < 0.001), and we also observed increased plasma fibrinogen levels, including the recovery of the ~54 kDa band that corresponds to the fibrinogen β chain (Fig. 6). EDTA therefore outperforms the heterologous antivenom combinations tested in terms of survival and survival times (vs. SAIMR boomslang, *P* = 0.002; vs. anti-ecarin, *P* = 0.005) and exhibits equivalence with the gold-standard species-specific antivenom EchiTAbG (Fig. 6). These observations underscore the key role of SVMPs in the overall toxicity of *E. ocellatus* venom.

## Discussion

Snake venoms consist of variable mixtures of bioactive proteins with distinct antigenic signatures, which undermine the generation of a single “universal” therapy to treat all snakebite victims. While certain pathologies, such as coagulopathy, can be underpinned by few toxins targeting specific physiological targets (e.g. *R. subminiatus* venom potently activating prothrombin), other snake venoms are more complex and their protein constituents act in a synergistic manner to perturb various host systems^45^, as evidenced by many of the procoagulant venoms in this study acting on multiple clotting factors (Fig. 2). Furthermore, many venoms also cause other pathologies in conjunction with coagulopathy, notably haemorrhage and/or myotoxicity^14,18,31^, and this combined action can greatly increase the risk of fatality.

Despite the therapeutic challenges associated with inter-specific variation in venom composition (i.e. the limited paraspecific efficacy of antivenom), we have demonstrated here that monospecific antivenoms and toxin-specific antibodies are capable of recognising various toxins found in the venom of geographically and phylogenetically distinct species (see EchiTAbG panel in Fig. 3) and neutralising certain clinically relevant venom activities in vitro (Figs. 1b, 5). Furthermore, this unexpected level of paraspecificity can also result in the neutralisation of phylogenetically distinct snake venoms in vivo (Fig. 6). In this study, we observed extensive similarities in the venom composition of the saw-scaled viper (*E. ocellatus*) and the boomslang (*D. typus*); two advanced snakes that separated over 54 million years ago and appear to have converged upon similar venom phenotypes^3,32,33^. We have demonstrated that both venoms contain abundant amounts of SVMPs that can be recognised and neutralised by heterologous antivenoms, resulting in increased survival times and parameters of coagulation in pre-clinical models of envenoming (Figs. 4–6).

These data suggest that while “universal antivenoms” might be very challenging at this time, “pathology-specific” (e.g. anti-coagulopathy, anti-haemorrhage) antivenoms, which target related toxin families found in diverse taxa that are responsible for causing life-threatening haemotoxic pathologies, seem technically achievable. Thus, despite extensive toxin variation, haemotoxic snake venoms predominately rely on the presence of relatively few toxin families (e.g. SVMPs, serine proteases, phospholipases A_2_, C-type lectins and/or disintegrins)^17,18^, which although expressed in multiple isoforms, typically share structural similarities. We therefore believe that informed choices of immunogens, whether via the selection of the most appropriate venoms or via purification or synthetic preparation of key pathogenically proven toxins (or chimeric fragments thereof)^46–48^ conserved across species, are likely to yield antibodies with far superior paraspecific neutralising capabilities than the highly promising results observed here with conventional antivenoms.

While providing important information strengthening the argument for the production of new types of antivenom for treating snakebite, our results also have direct implications for the current treatment of snakebite, as we demonstrated that anti-*Echis* antibodies (EchiTAbG antivenom) provide significant protection against the toxic effects of boomslang venom in vivo. Both saw-scaled vipers (*Echis* spp.) and boomslangs (*D. typus*) cause life-threatening venom-induced consumption coagulopathy in envenomed victims^14^. However, while saw-scaled vipers likely account for more snakebite deaths worldwide than any other group of snakes^30^, bites and deaths by boomslangs are far less common due to the arboreal nature of this species^31^. Nonetheless, South Africa Vaccine Producers manufacture the SAIMR boomslang antivenom to treat such envenomings, but the availability of this product is extremely limited and it is very expensive outside of southern Africa (in late 2016 we were quoted US$6050 per vial), making it completely unobtainable to most impoverished African snakebite victims. Our preclinical results here suggest that in the absence of the SAIMR boomslang antivenom (whether due to unavailability or affordability), an anti-saw-scaled viper antivenom could potentially be a useful clinical tool capable of neutralising some boomslang venom toxins and, perhaps, delaying the onset of severe signs of envenoming. While clinical observations will be required to validate the potential benefit of using saw-scaled viper antivenom to treat boomslang bites, the absence of alternative treatment strategies in rural tropical Africa strongly advocate for its trial in cases of severe, life-threatening, envenoming.

Enzyme inhibitors potentially offer an alternative (or adjunct), non-antibody based, future treatment for snakebite^49^. For example, recent studies have demonstrated that Batimastat, a peptidomimetic hydroxamate metalloproteinase inhibitor, abrogates the main local and systemic effects induced by *E. ocellatus* venom in vivo, even in conditions where the inhibitor is administered after envenoming^50^. Similarly, varespladib, a phospholipase A_2_ inhibitor, offered varying degrees of in vivo protection and rescue against venom-induced lethality when co-administered with, and following administration of, venom from *Micrurus fulvius* and *Vipera berus*^51^. Herein we demonstrated that pre-incubation of venom with EDTA protected mice from lethality caused by one of the world’s most medically important snake species, *E. ocellatus*. These data represent the first evidence to our knowledge of metal chelators preventing venom-induced murine lethality in vivo. In combination with prior reports of EDTA neutralising specific markers of haematopathology caused by snake venoms^41–43^, these results suggest that metal chelation could be an effective means to inhibit zinc-dependant SVMP toxins in vivo. Although EDTA’s potential chelation of circulating calcium may impact upon its clinical utility, some EDTA salts, such as CaNa_2_EDTA, have previously been used in humans for the treatment of heavy metal poisoning, though more recently they have been substituted by other chelating agents^52^. Nonetheless, this pilot study advocates that enzyme inhibitors and metal chelating agents warrant extensive re-exploration for their potential to deliver inexpensive, enzyme family-specific, and thus snake paraspecific, inhibitory actions of benefit for neutralising snake venom toxins in clinical settings. Consequently, future work in our laboratory will focus on assessing the paraspecific venom neutralising capability of various inhibitory agents, following the promising findings obtained here. In addition, we will seek to address the inherent limitation of pre-incubating venom and inhibitors/antivenoms in preclinical studies. While this approach is in line with World Health Organization and International Pharmacopoeia guidelines^53^, and is undoubtedly an important first step for the characterisation of venom neutralisation in vivo, this approach does not reflect the clinical scenario where envenoming precedes treatment. Thus, in the future we will also seek to undertake preclinical efficacy studies where treatment follows the administration of venom.

In summary, our study emphasises that snake venoms are mixtures of various toxins that can work synergistically to perturb physiological systems, such as the coagulation cascade. Identifying the toxins likely responsible for causing pathologies like venom-induced consumption coagulopathy and the physiological targets that they interact with, provides a sound basis for rationally testing the paraspecific neutralising capability of existing antivenoms. Here, we have demonstrated that antivenoms can work in a previously unrecognised paraspecific manner. It is therefore apparent that obtaining knowledge surrounding venom composition has the potential to identify unexpected therapeutic benefits of existing antivenoms. These results also offer much hope to the future design of more paraspecifically effective, toxin-targeted antivenoms, whereby cross-neutralisation of different snake venoms might be achieved economically and without greatly increasing therapeutic doses, and therefore compromising the affordability and safety of these products to impoverished snakebite victims. While antibody-based therapies will undoubtedly remain the mainstay of snakebite treatment for the foreseeable future, our results also strongly justify a thorough re-assessment of the potential generic utility of enzyme inhibitors and metal chelators as adjunct therapies for snake envenoming.

## Methods

### Biological samples

A total of 57 snake venoms were used in this study (Supplementary Table 1). These venoms were sourced from: (i) animals currently housed in the herpetarium of, and (ii) historical lyophilised venom samples stored long term at 4 °C in, the Alistair Reid Venom Research Unit, Liverpool School of Tropical Medicine and (iii) Latoxan (France). The venoms used here represent snakes from every continent and from four advanced snake families and sub-families; the front-fanged, medically important Viperidae and Elapidae and the non-front fanged Colubrinae and Natricinae (Supplementary Table 1). All venoms were lyophilised and stored 4 °C before reconstitution in PBS buffer (pH 7.4) and short-term storage at −80 °C until use.

The commercial antivenoms used in this study were: (i) the monospecific anti-*E. ocellatus* antivenom “EchiTAbG^®^” (25 mg/ml) that is an ovine antivenom containing intact IgG immunoglobulins manufactured by MicroPharm Limited, UK, (ii) the monospecific anti-*D. typus* “SAIMR boomslang” antivenom (75 mg/ml) that is an equine antivenom containing F(ab′)_2_ immunoglobulin fragments manufactured by South African Vaccine Producers (SAVP), South Africa and (iii) the polyspecific anti-Australian elapid (anti-*O. scutellatus*, -*Pseudechis australis*, -*Notechis scutatus*, -*Pseudonaja textilis* and *-Acanthophis antarcticus*) “CSL polyvalent” antivenom (87.5 mg/ml) which is also equine F(ab′)_2_ and manufactured by Seqirus Pty Ltd (formally Commonwealth Serum Laboratories), Australia. Antivenom concentrations were determined using a NanoDrop (Thermo Scientific) with the protein A280 method using the in-built IgG mass extinction coefficient.

Anti-ecarin IgG antibodies were generated from serum previously collected from rabbits immunised with ecarin (Pentapharm, Basel, Switzerland)^24^. To purify IgG, we used the caprylic acid precipitation method previously described^8^, which involved the addition of caprylic acid (5% volume), vigorous stirring for 2 h, before centrifugation, dialysis overnight in PBS and formulation of antibodies to a concentration of 25 mg/ml. We repeated this process to generate control IgG immunoglobulins from normal non-immunised sheep, horse (both sourced from Sigma-Aldrich, Gillingham, UK) and rabbit^54^ serum.

### Plasma assays

*MCD-P screening*: To determine which venoms exhibited procoagulant activity without the addition of cofactors (e.g. calcium), each venom was screened in a modified version of the MCD-P assay^16^. We added 100 μg (2 mg/ml) of each venom to 200 μl of human citrated plasma (4% trisodium citrate, Sigma-Aldrich) in a glass test tube in triplicate, and then incubated the samples in a water bath at 37 °C for 5 min. Those that produced a well-defined fibrin clot were selected for further analysis.

*MCD-P*: Each of the 18 venoms exhibiting procoagulant activity (defined by clot formation) were subjected to traditional MCD-P assays^16^ to determine the quantity of each venom required to clot 200 μl of human plasma in 60 s. Varying doses of each venom (made to 50 μl in PBS) were added to 200 μl of human plasma, incubated at 37 °C and time-monitored for clot formation. The mean coagulation time of triplicate results for each venom dose were plotted against dose, and the dose resulting in a clot at 60 s was calculated using the equation of the line of best fit.

To reconstruct the evolutionary history of procoagulant venom function we manually constructed a species tree from previously published phylogenies^55–58^ for the procoagulant species determined in this study. We then assigned binary character states to each species based on whether the venom coagulated human plasma in the MCD-P screening assay. We reconstructed ancestral character states by tracing the character history using the likelihood ancestral states analysis in Mesquite^59^, and overlaid this information, including proportional likelihoods for the “procoagulant character state” at ancestral nodes, onto the species phylogeny.

*Neutralising MCD-P*: We tested the neutralising capability of the various antivenoms (EchiTAbG, SAIMR boomslang, CSL polyvalent) and antibodies (anti-ecarin and normal horse, sheep and rabbit IgG) in a modified version of the assay described above. The MCD dose of each venom was incubated at 37 °C for 30 min with varying doses of each antivenom/antibody preparation (0.1, 1, 10 and 30 μl) in a total incubation volume of 50 μl. After incubation, the venom/antibody mixture was added to 200 μl of human plasma, incubated at 37 °C and monitored for clot formation, as described above. If the plasma did not clot within 120 s (a robust endpoint representing two times the MCD-P coagulation time), the antivenom was deemed to neutralise procoagulant venom activity.

*MCD-P with factor-deficient plasma*: We tested the capability of each of the 18 procoagulant venoms to clot human plasma deficient in Factor X or prothrombin in modified MCD-P assays. For each venom we used 50 μl doses consisting of one and ten times the MCD dose determined above, and added these to 200 μl of human plasma deficient in either Factor X or prothrombin (Haematological Technologies, Inc.). We then incubated the samples at 37 °C for 5 min and monitored clot formation, as described above.

### Degradation SDS-PAGE gel electrophoresis

We used SDS-PAGE gel electrophoresis to determine whether Factor X, prothrombin or fibrinogen were cleaved (activated/degraded) by the 18 procoagulant snake venoms. For each venom we performed the following experiment with lanes containing: 5 µg of the relevant clotting factor, 10 µg of the relevant clotting factor; 5 µg of venom; 5 µg of clotting factor and 5 µg venom, and 10 µg of clotting factor and 5 µg venom. All samples were prepared and incubated for 60 min at 37 °C before the addition of a reduced protein loading buffer at a ratio of 1:1. Samples were loaded onto ten-well Mini-PROTEAN TGX precast AnykD gels (Bio-Rad) alongside a protein marker (Broad Range Molecular Marker, Promega) and run at 100 V for 60 min using a Mini-PROTEAN Tetra System (Bio-Rad). Resulting gels were stained with coomassie brilliant blue overnight and then destained (4.5:1:4.5 methanol:acetic acid:H_2_O) for visualisation.

We repeated these assays using *E. ocellatus* and *D. typus* venom, and the SVMP toxin ecarin, with prothrombin, but in the presence of the metal chelator EDTA (E6758, Sigma-Aldrich). The gel lanes contained: 5 µg of clotting factor, 10 µg venom, 5 µg of clotting factor and 10 µg venom, and then 5 µg of clotting factor and 10 µg venom in the presence of ten-fold molar dilutions of EDTA, starting at 10 mM and finishing at 1 µM. Gel electrophoresis was performed as described above, with the exception that venom and EDTA were mixed and incubated at 37 °C for 30 min prior to the addition of prothrombin.

### Chromogenic enzyme assay

*Venom activity*: We developed a chromogenic assay using the thrombin-specific chromogenic substrate S-2238 (Cambridge Biosciences) to measure the thrombin-like enzyme activity, prothrombin activating activity and Factor X activating activity of each of the 18 procoagulant snake venoms. To measure thrombin-like enzyme activity, we plated the following reaction for each venom in triplicate onto 96-well plates and measured changes in absorbance at 405 nm every 3 min for 21 min using an LT-4500 microplate reader (LabTech): 93 µl Tris buffer (50 mM Tris, 175 mM NaCl, pH 7.4), 1 µl of venom (1 µg), 5 µl PBS and 1 µl of 3 mM S-2238 chromogenic substrate. A negative control, consisting of no venom (93 µl Tris buffer, 1 µl substrate, 6 µl PBS), was used in every experiment. A positive control, consisting of 1 µl of 0.1 units/µl of thrombin (Sigma-Aldrich) instead of venom, was used to validate the assay. To measure the prothrombin activating activity, we repeated the experiment above using 1 µl of prothrombin (200 ng; Haematological Technologies, Inc.) and 4 µl PBS. To measure Factor X activating activity, we repeated the prothrombin experiment with the addition of 1 µl of Factor X (Haematological Technologies, Inc.) and 3 µl PBS. Mean measures of absorbance were plotted against time to compare venom activity with baseline (negative controls) and positive control readings. We then subtracted the mean of the relevant negative control readings from the venom readings and re-plotted the triplicate readings. To calculate prothrombin activation we subtracted these readings from those obtained in the presence of prothrombin, and for Factor X activation, we subtracted the prothrombin readings from those obtained when using Factor X and prothrombin. For all data sets we then calculated the areas under the curve and the standard error of the mean (of total peak areas) using default parameters in GraphPad Prism5.

*Neutralisation of in vitro prothrombin activation by antivenom*: Due to the high level of prothrombin activation caused by *E. ocellatus* and *D. typus* venoms, we repeated the prothrombin chromogenic assays for these two venoms in the presence of antivenoms/antibodies and EDTA. Using the method described above, we used the EchiTAbG, SAIMR boomslang and CSL antivenoms, and anti-ecarin antibodies, at standardised doses of 150 µg and varying concentrations of EDTA (tenfold molar dilutions of 10 mM to 1 µM) in place of PBS. We used the method described above with the exception of pre-incubating the venom (1 µg) and antibody or EDTA samples with the Tris buffer at 37 °C for 30 min, prior to the addition of prothrombin (200 ng) and the S-2238 chromogenic substrate (1 µl of 3 mM). As above, we repeated all experiments in the absence of venom to generate baseline negative controls, which were subtracted from venom readings. For statistical analysis, we used mean endpoint absorbances in a one-way ANOVA with Bonferroni adjustment followed by Tukey’s post hoc test, in programming language R (version 3.3.3, R Foundation for Statistical Computing, 2017), with a 95% family-wise confidence level.

### Immunological analyses

*1D SDS-PAGE and western blotting*: One-dimensional (1D) SDS-PAGE gel electrophoresis was performed for the 18 venoms as described earlier. We used 10 µg of each venom with a 1:1 ratio of reduced protein loading buffer, incubated the samples at 100 °C for 10 min and loaded them onto ten-well Mini-PROTEAN TGX precast AnykD gels (Bio-Rad), before running them at 100 V for 60 min using a Mini-PROTEAN Tetra System (Bio-Rad). We used a Trans-Blot Turbo Transfer System (Bio-Rad) to transfer proteins on to 0.45 µm nitrocellulose membranes. Following confirmation of successful protein transfer by reversible Ponceau S staining, the membranes were incubated overnight in 5% non-fat milk in TBST buffer (0.01 M Tris-HCl, pH 8.5; 0.15 M NaCl; 1% Tween 20) and then washed six times in TBST over 90 min before incubation overnight at 4 °C with the different primary antibodies (EchiTAbG, SAIMR boomslang and CSL antivenoms and anti-ecarin antibodies) diluted 1:5000 in 5% non-fat milk in TBST. Blots were washed again and incubated for 2 h at room temperature with horseradish peroxidase-conjugated secondary antibodies (donkey anti-sheep for EchiTAbG; rabbit anti-horse for SAIMR boomslang and CSL polyvalent; goat anti-rabbit for anti-ecarin; all Sigma-Aldrich) diluted 1:2000 in PBS. After a final TBST wash, immunoblots were visualised with the addition of DAB substrate (50 mg 3,3-diaminobenzidine, 100 ml PBS and 0.024% hydrogen peroxide; Sigma, UK) for 10 s.

*2D SDS-PAGE and western blotting*: We performed two dimensional (2D) SDS-PAGE gel electrophoresis experiments using *E. ocellatus* and *D. typus* venoms and used western blotting to visualise venom protein−antibody interactions with the EchiTAbG and SAIMR boomslang antivenoms and anti-ecarin antibodies. For each gel, 0.5 mg of venom was prepared for 2D gel electrophoresis using the ReadyPrep™ 2-D Cleanup Kit for isoelectric focusing (IEF) (Bio-Rad) as per the manufacturer’s instructions. Cleaned-up venom samples were then applied to 7 cm, pH 3–10, non-linear IPG strips (Bio-Rad) using the ReadyPrep™ 2-D starter kit (BioRad), as per the manufacturer’s instructions, and re-hydrated overnight at room temperature. After re-hydration, IEF was performed using a PROTEAN^®^ IEF Cell (Bio-Rad) with the manufacturer’s standard electrophoresis protocol for 7 cm IPG strips (default cell temperature = 20 °C; maximum current 50 Ua/strip; voltage = 250 V with linear ramp for 20 min; 4000 V with linear ramp for 2 h; 4000 V with rapid ramp for 10,000 V-hr). After IEF, IPG strips were equilibrated (as per the ReadyPrep™ 2-D starter kit) and loaded onto Mini-PROTEAN TGX AnyKd precast gels (Bio-Rad) and run at 200 V for 30 min. Gels were then either stained with coomassie brilliant blue or used in western blots. We undertook western blotting as described above, with the exception that we standardised the primary antibodies to 5 µg/ml in 5% non-fat milk in TBST and added the secondary antibodies at 1:5000 dilutions in TBST.

*Antivenomics*: A second-generation antivenomics approach^36^ was applied to examine the immunoreactivity of EchiTabG, SAIMR boomslang and anti-ecarin antivenom/antibodies against *E. ocellatus* and *D. typus* venoms. To prepare the antivenom affinity columns, 350 μl of CNBr-activated Sepharose™ 4B matrix (GE Healthcare) was packed in a Pierce centrifuge column and washed with 10× matrix volume of cold 1 mM HCl, followed by 2× matrix volume of 0.2 M NaHCO_3_, 0.5 M NaCl, pH 8.3 (coupling buffer) to adjust the pH of the column to 7.0–8.0. Antivenoms were dialysed against MilliQ water, lyophilised and reconstituted in coupling buffer. The concentrations of the antivenom stock solutions were determined spectrophotometrically using a 1 cm light path length cuvette and an extinction coefficient at 280 nm of 1.36 for a 1 mg/ml concentration of antivenom. Four milligrams of EchiTabG, SAIMR boomslang and anti-ecarin antivenoms/antibodies were dissolved in a half matrix volume of coupling buffer and incubated with the matrix for 4 h at room temperature. Antivenom coupling yields, estimated measuring A_280_ before and after coupling of the antivenom, were 100% for all antibody preparations. After coupling, remaining reactive matrix groups were blocked at room temperature for 4 h with 350 μl of 0.1 M Tris-HCl, pH 8.5. Affinity columns were then alternately washed with 3× 350 μl volumes of 0.1 M acetate containing 0.5 M NaCl, pH 4.0–5.0, and 3× 350 μl volumes of 0.1 M Tris-HCl, pH 8.5. This procedure was repeated six times. The columns were then equilibrated with five volumes of working buffer solution (PBS: 20 mM sodium phosphate, 135 mM NaCl, pH 7.4).

For the immunoaffinity assay, increasing amounts (50, 75, 100 and 125 μg) of *E. ocellatus* and *D. typus* venoms were dissolved in half matrix volumes of PBS and incubated with the affinity matrix for 1 h at room temperature using an orbital shaker. As specificity controls, 350 μl of CNBr-activated Sepharose™ 4B matrix alone (mock matrix) or with 4 mg of control IgG isolated from the plasma of non-immunised horses (gifted by Instituto Clodomiro Picado, Costa Rica) were incubated with venom and the control columns developed in parallel to the immunoaffinity experiment. Non-retained fractions were collected with 5× matrix volumes of PBS, and the immunocaptured proteins eluted with 5× matrix volumes of elution buffer (0.1 M glycine-HCl, pH 2.0) and neutralised with 175 μl of 1 M Tris-HCl, pH 9.0. The flow-through and the immunocaptured venom fractions were lyophilised, reconstituted in 40 μl MilliQ water, and fractionated by reverse-phase HPLC using a Discovery^®^ BIO Wide Pore C_18_ (15 cm × 2.1 mm, 3 μm particle size, 300 Å pore size) column and an Agilent LC 1100 High Pressure Gradient System equipped with a DAD detector. The RP-HPLC column was run at flow rate of 0.4 ml/min and proteins eluted with a linear gradient of 0.1% TFA in MilliQ water (solution A) and 0.1% TFA in acetonitrile (solution B), isocratically with 5% solution B for 1 min, followed by linear gradients of 5–25% B for 5 min, 25–45% B for 35 min, and 45–70% B for 5 min. Proteins were detected at 215 nm with a reference wavelength of 400 nm.

The fraction of non-immunocaptured protein “i” was estimated as the relative ratio of the chromatographic areas of same protein recovered in the non-retained (NRi) and retained (Ri) affinity chromatography fractions using the equation %NRi = 100−[(Ri/(Ri + NRi)) × 100]^36^. For SVMPs, owing to their high affinity of binding, the percentage of non-immunocaptured SVMP“i” (% NR_SVMP“i”_) was calculated as the ratio between the chromatographic areas of the same SVMP peak recovered in the non-retained fraction (NR_SVMP“i”_) and in the injected venom (V_SVMP“i”_), using the equation %NR_SVMP“i”_ = (NR_SVMP“i”_/V_SVMP“i”_) × 100. Identification of the immunocaptured and the non-immunoretained venom components was inferred by comparing the reverse-phase chromatographic separations to our previously characterised proteomic profiles of *E. ocellatus*^32^ and *D. typus* venoms^33^.

### In vivo venom neutralisation

*Venom lethality*: All in vivo animal experimentation was conducted using protocols approved by the Animal Welfare and Ethical Review Boards of the Liverpool School of Tropical Medicine and the University of Liverpool, and performed in specific pathogen-free conditions under licenced approval of the UK Home Office, in accordance with the Animal [Scientific Procedures] Act 1986 and institutional guidance on animal care. Experimental design was based upon refined WHO-recommended protocols^8,11,16,53^. We first determined the median lethal dose (venom LD_50_) of *E. ocellatus* and *D. typus* venom, as previously described^8^. Groups of five male 18–22g CD-1 mice (Charles River, UK) received varying doses of each venom in 100 μl PBS via intravenous (tail vein) injection, and after 6 h surviving animals were recorded. Animals were monitored for the duration of the experiment and euthanised upon observation of humane endpoints (external signs of haemorrhage, seizure, pulmonary distress, paralysis). The amount of venom that causes lethality in 50% of the injected mice (the LD_50_) and the 95% confidence intervals were calculated using probit analysis^60^.

*Venom neutralisation*: Next we used 2.5 × LD_50_ doses of each venom (*E. ocellatus* 17.85 μg; *D. typus* 22.29 μg) in modified versions of antivenom effective dose (ED_50_) neutralisation experiments^61^. As above, groups of five male CD1 mice (18–22g) received experimental doses, which consisted of either: (i) venom only, (ii) venom and antibodies (7.5 mg (375 μg/g) of EchiTAbG, SAIMR boomslang, CSL polyvalent or anti-ecarin antibodies), (iii) venom and EDTA (100 μg (5 μg/g) of EDTA, pH 7.1), (iv) antibodies only (7.5 mg), (v) EDTA only (100 μg) or (vi) PBS only (normal mouse control). Where required (for EchiTAbG and anti-ecarin), antibodies were first concentrated using 50,000 molecular weight cutoff Amicon Ultra Centrifugal filters (Sigma-Aldrich), as per the manufacturer’s instructions. The EDTA dose was conservatively selected based on prior reports demonstrating that daily doses of 15–60 μg/g for 14 days were non-toxic in mice^44^. All experimental doses were prepared to a volume of 200 μl in PBS and incubated at 37 °C for 30 min prior to their intravenous injection. Animals were monitored for 6 h as described for the LD_50_ experiments and deaths, time of death and survivors recorded, where “deaths/time of death” actually represents the implementation of euthanasia based on defined humane endpoints.

*Murine plasma assays*: Immediately following euthanasia, blood was collected via cardiac puncture and added to citrated tubes containing 3.2% buffered sodium citrate to prevent coagulation. Blood samples were then spun at 2000 × *g* for 20 min to generate plasma samples which were stored immediately at −80 °C until further use. The concentrations of circulating TAT in murine plasma were measured as described previously^62^ using the Mouse Thrombin-Antithrombin Complexes ELISA Kit (ab137994; Abcam), per the manufacturer’s instructions. We used plasma sourced from three individuals from each experimental group and statistically analysed the data as described earlier (one-way ANOVA with Tukey’s HSD post hoc test in R).

We next used 1D SDS-PAGE and western blotting to analyse the collected plasma samples. For each treatment group we used pooled plasma (10 µl per individual; *n* = 5). For 1D SDS-PAGE, pooled plasma were diluted 1:50 in PBS before the addition of reduced protein loading buffer (1:1 ratio) and boiled for 10 min. Control samples consisted of 1 µg of either Factor X, fibrinogen, prothrombin or thrombin and were prepared 1:1 with protein loading buffer as above. Samples were loaded onto 15-well Mini-PROTEAN TGX precast AnykD gels (Bio-Rad) and electrophoresis and protein staining carried out as described above. For immunoblotting, 1D SDS-PAGE gels were run in an identical manner, except that pooled plasma was diluted 1:20 in PBS. The protein samples were transferred to nitrocellulose membranes, with blocking and washing performed as described earlier. Membranes were incubated in either 1:1000 dilution of polyclonal rabbit anti-prothrombin primary antibody or 1:2000 dilution of polyclonal rabbit anti-fibrinogen primary antibody (both Abcam, UK) in 5% non-fat milk TBST, and incubated for 1 h at room temperature with gentle shaking. Secondary antibody incubations were performed using horseradish peroxidase-conjugated goat anti-rabbit (Sigma-Aldrich) diluted 1:1000 (anti-prothrombin blots) or 1:2000 (anti-fibrinogen blots) in PBS and incubated as above for 1 h. Immunoblots were visualised as described earlier. We were unable to use plasma sourced from mice receiving anti-ecarin antibodies in these experiments, as these antibodies were generated in rabbits and thus cross-reacted extensively in a non-specific manner with the anti-rabbit secondary antibody.

### Data availability

The data sets generated and analysed during the current study are available from the corresponding author on request.

## Electronic supplementary material

Supplementary Information
