# Supplementary material for: The paraspecific neutralisation of snake venom induced coagulopathy by antivenoms
Source: Commun Biol. 2018 Apr 19;1:34. doi: 10.1038/s42003-018-0039-1 (PMC6123674; doi:10.1038/s42003-018-0039-1)
Supplement: Supplementary file 1 — Supplementary Information [file 42003_2018_39_MOESM1_ESM.pdf]

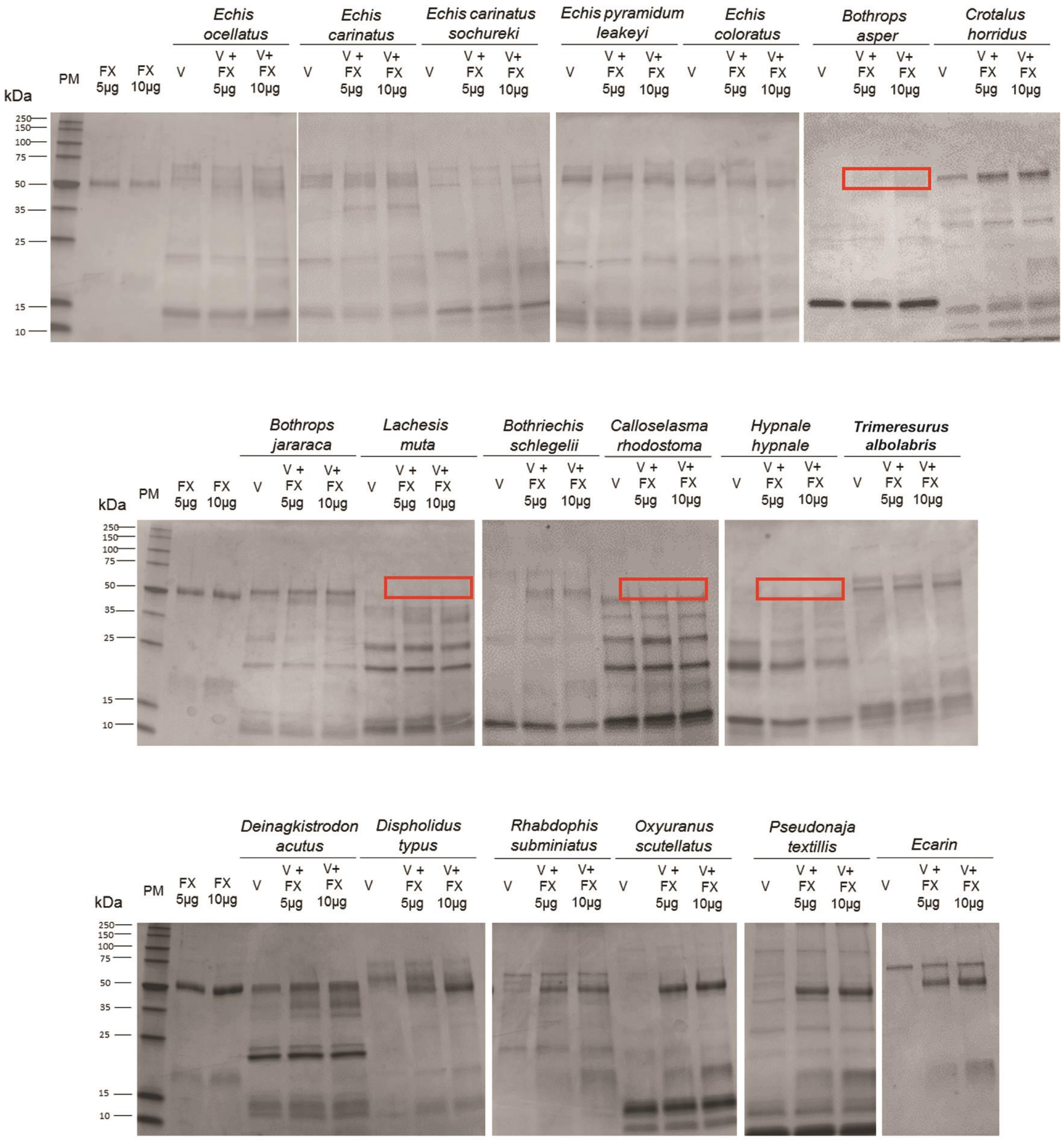

Supplementary Figure 1. The cleavage of Factor X by the 18 procoagulant snake venoms used in this study. Factor X (FX) was incubated with and without venom (V) at different concentrations (5 µg and 10 µg) and analysed by reduced gel electrophoresis. The Factor X control is displayed on the left of each row (for display purposes these controls and the protein marker have been spliced from the other gels in each row). Red boxes indicate definitive depletion of Factor X, indicative of cleavage.

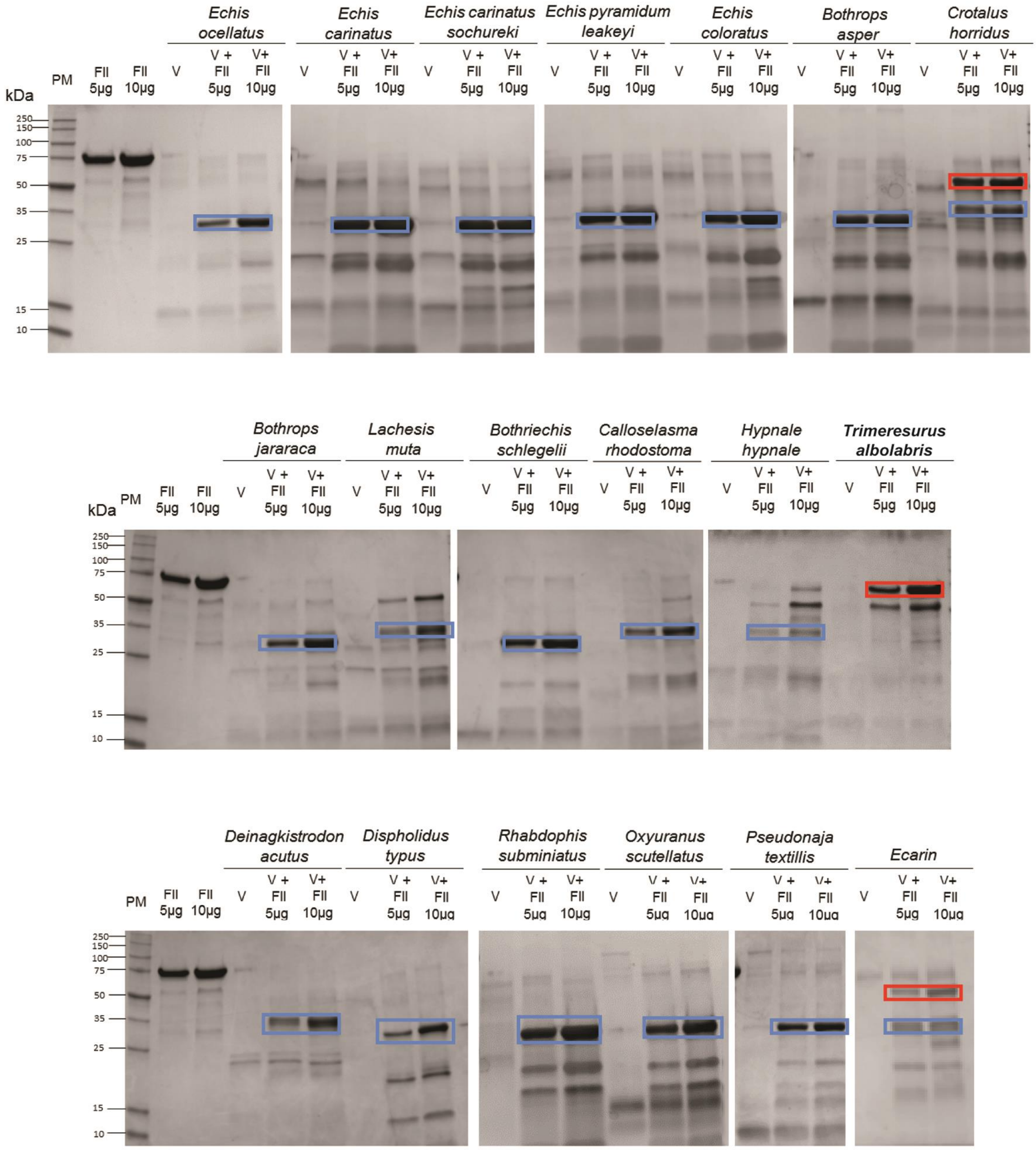

Supplementary Figure 2. The cleavage of prothrombin by the 18 procoagulant snake venoms used in this study. Prothrombin (FII) was incubated with and without venom (V) at different concentrations (5 μg and 10 μg) and analysed by reduced gel electrophoresis. The prothrombin control is displayed on the left of each row (for display purposes these controls and the protein marker have been spliced from the other gels in each row). Boxes indicate clearly defined cleavage products produced: red boxes correspond to meizothrombin (48 kDa) and blue boxes correspond to thrombin (36.7 kDa) or the β-chain of thrombin (31 kDa).

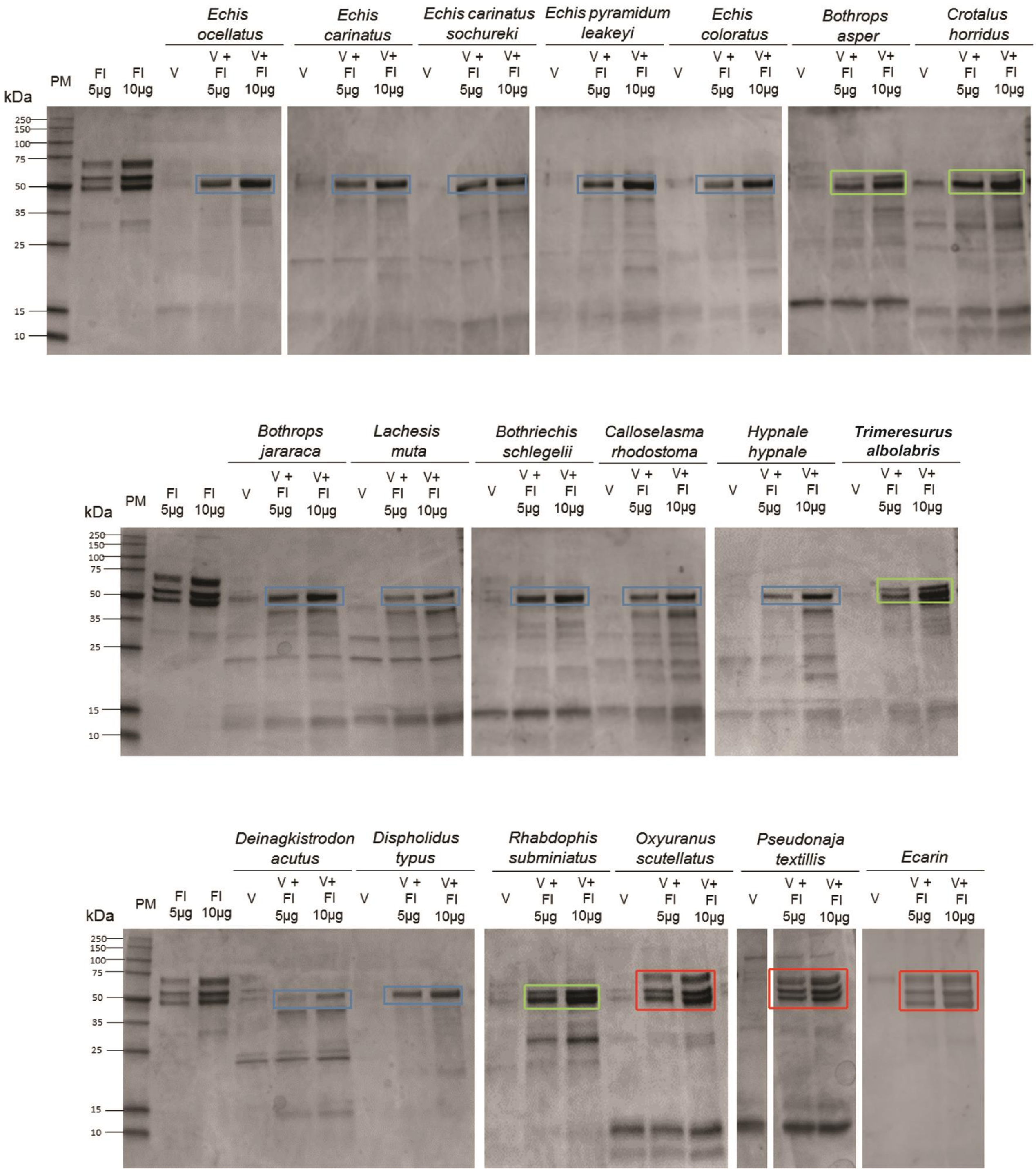

Supplementary Figure 3. The cleavage of fibrinogen by the 18 procoagulant snake venoms used in this study. Fibrinogen (FI) was incubated with and without venom (V) at different concentrations (5 µg and 10 µg) and analysed by reduced gel electrophoresis. The three chains of fibrinogen (α-chain ~63.5 kDa; β-chain ~56 kDa; γ-chain ~47 kDa) are evident in the controls on the left hand side of each row (for display purposes these controls and the protein marker have been spliced from the other gels in each row). Red boxes indicate no apparent cleavage of fibrinogen, green boxes indicate cleavage of the α-chain only and blue boxes indicate cleavage of both α- and β-chains.

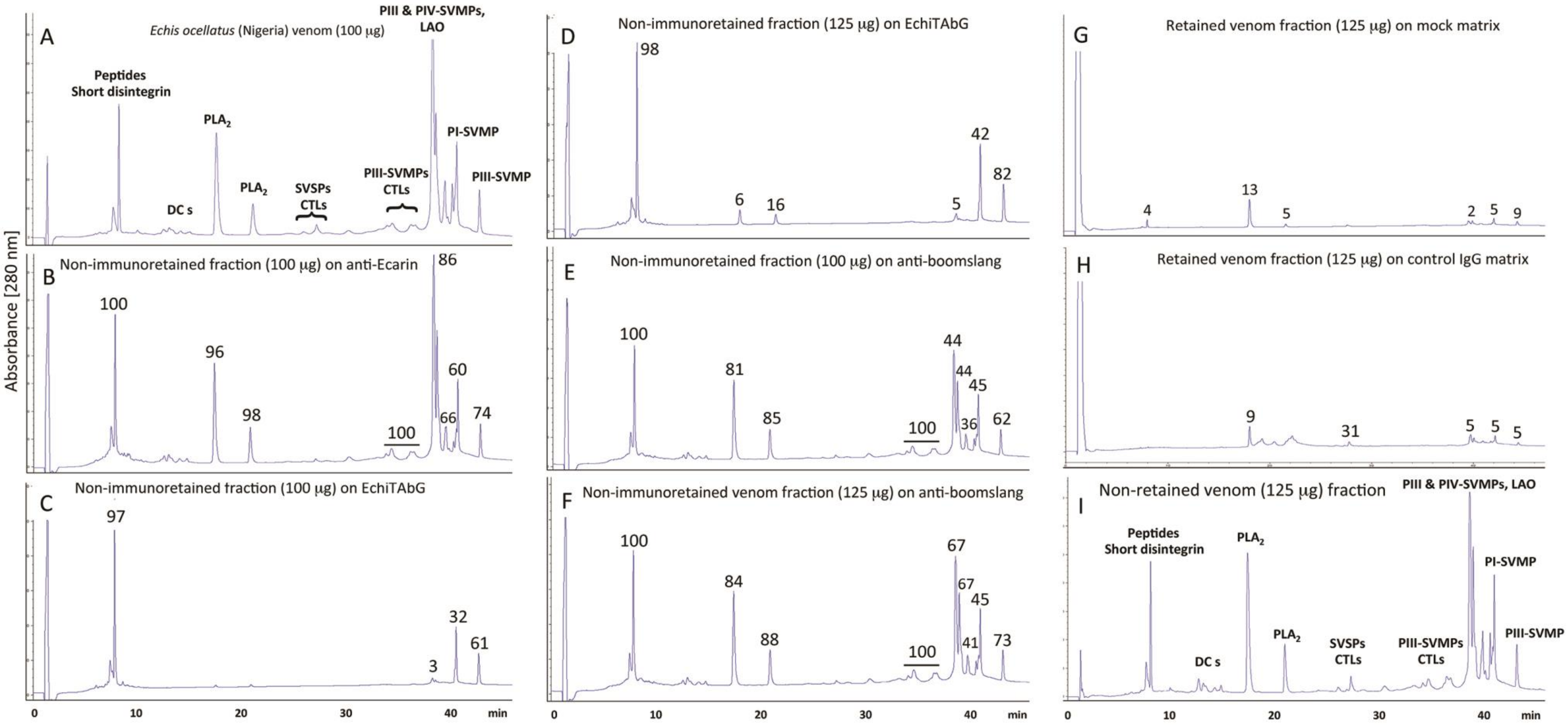

Supplementary Figure 4. Antivenomic analysis of bound (immunoretained) and unbound (non-immunoretained) toxins in the venom of the saw-scaled viper (*Echis ocellatus*) when exposed to species-appropriate (EchiTAbG) and -inappropriate (anti-boomslang [SAIMR boomslang] and anti-ecarin) antivenoms. Numbers above peaks represent the percentage of each major venom peak that remains following binding interactions. See also Figure 4.

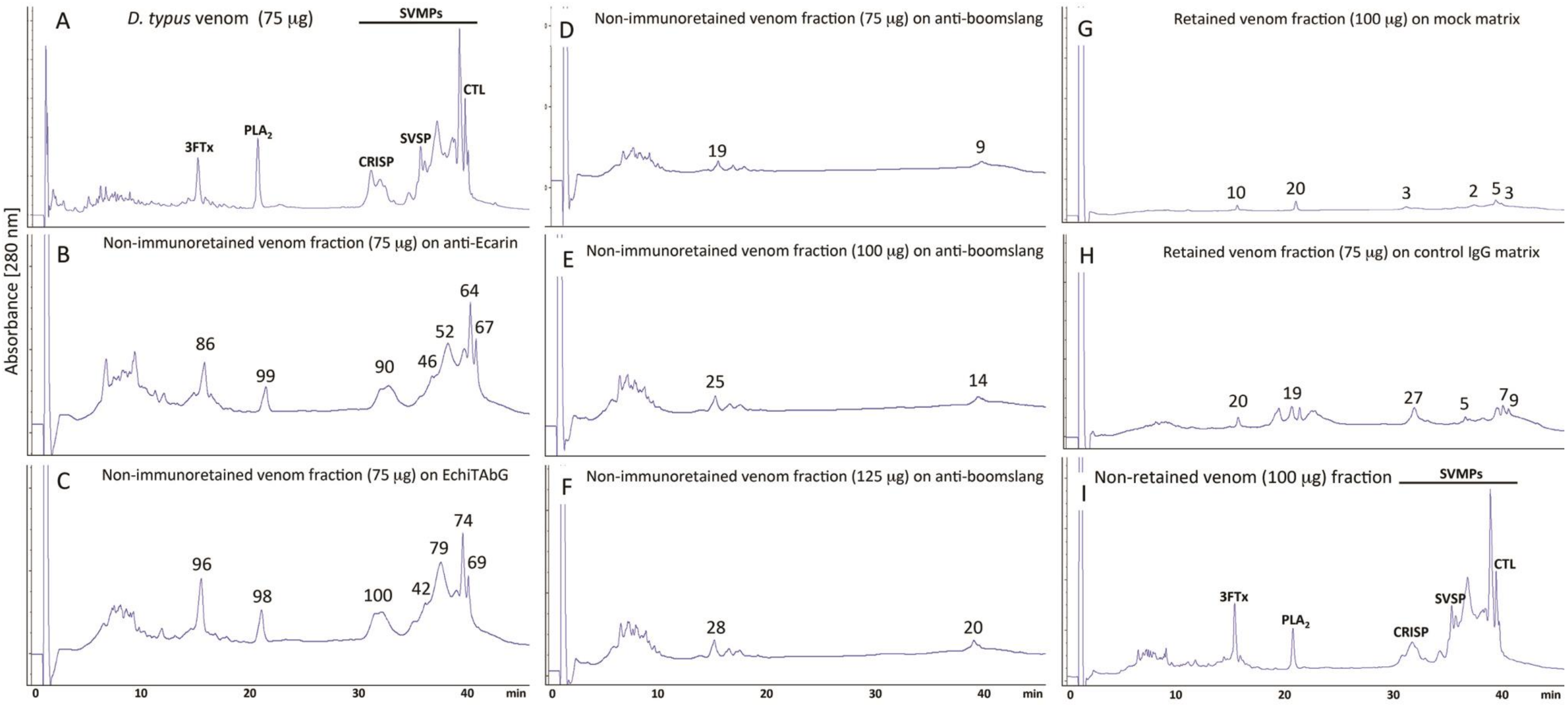

Supplementary Figure 5. Antivenomic analysis of bound (immunoretained) and unbound (non-immunoretained) toxins in the venom of the boomslang (*Dispholidus typus*) when exposed to species-appropriate (anti-boomslang [SAIMR boomslang]) and -inappropriate (EchiTAbG and anti-ecarin) antivenoms. Numbers above peaks represent the percentage of each major venom peak that remains following binding interactions. See also Figure 4.

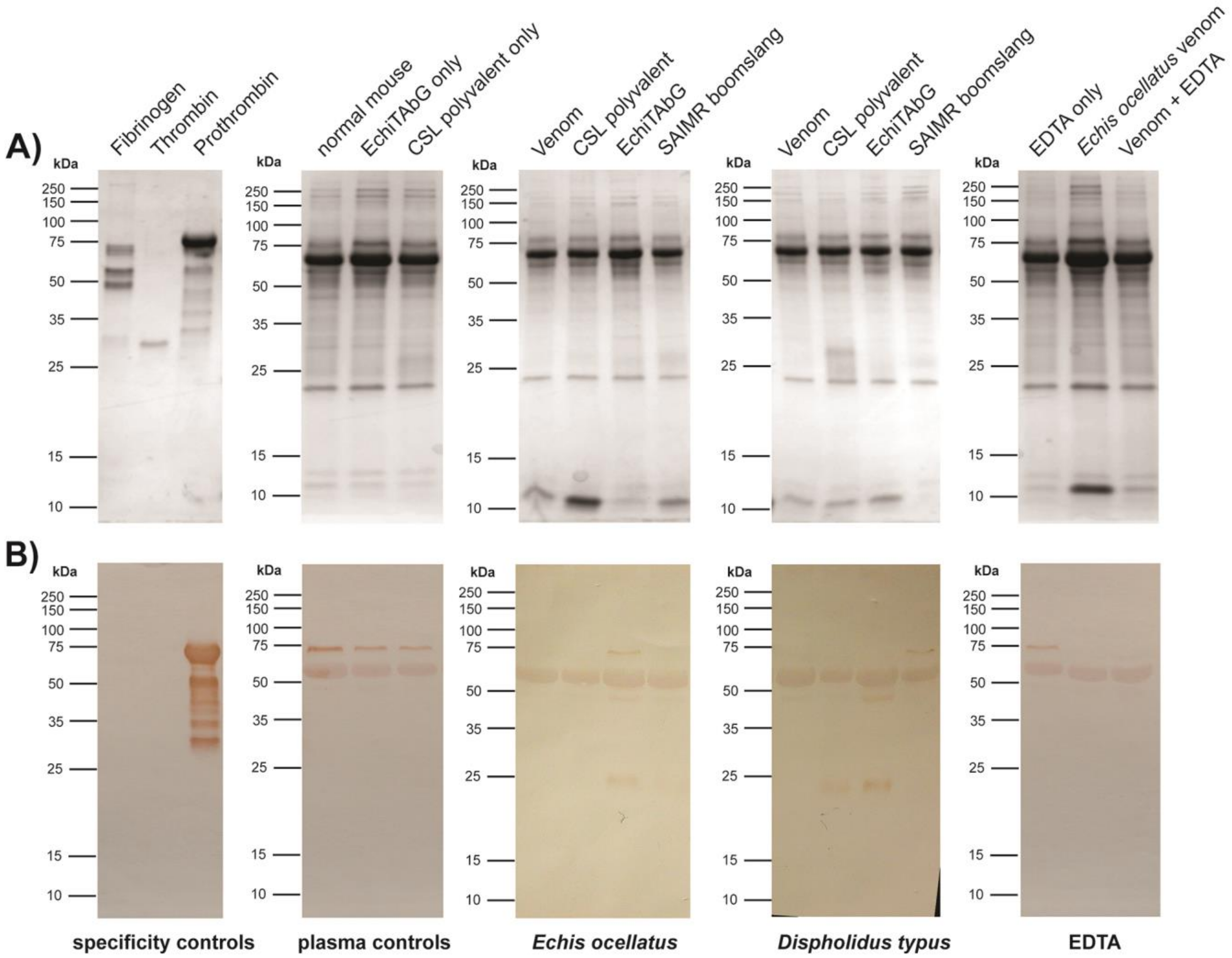

Supplementary Figure 6. Analysis of murine plasma obtained from the *in vivo* venom neutralisation study by SDS-PAGE gel electrophoresis and Western blotting. **A)** One dimensional SDS-PAGE analysis of pooled plasma (n=5) taken from each experimental group of mice challenged with venom and/or antivenoms/EDTA. **B)** Western blotting analysis of the same plasma with an anti-prothrombin antibody. Note that mice treated with anti-ecarin antibodies (raised in rabbits) were not included in this analysis as the anti-prothrombin antibody used was of rabbit origin and therefore the secondary, anti-rabbit antibody showed extensive cross-reactivity (i.e. loss of specificity).

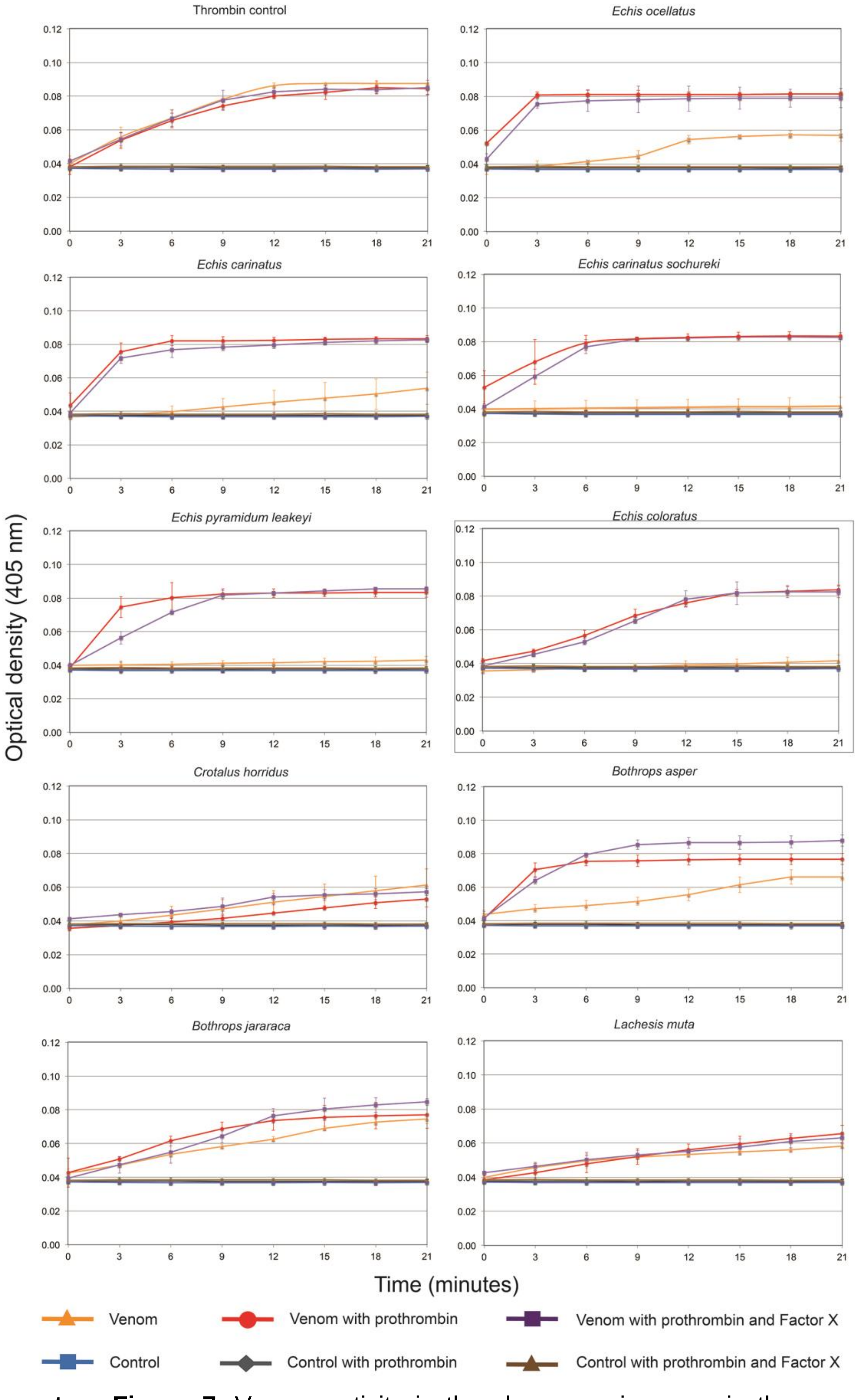

Supplementary Figure 7. Venom activity in the chromogenic assay in the presence of different clotting factors. Lines represent means of triplicate measurements and error bars represent SEMs. The lines displayed were used for the area under the curve analyses presented in Figure 2, following subtraction of appropriate control readings.

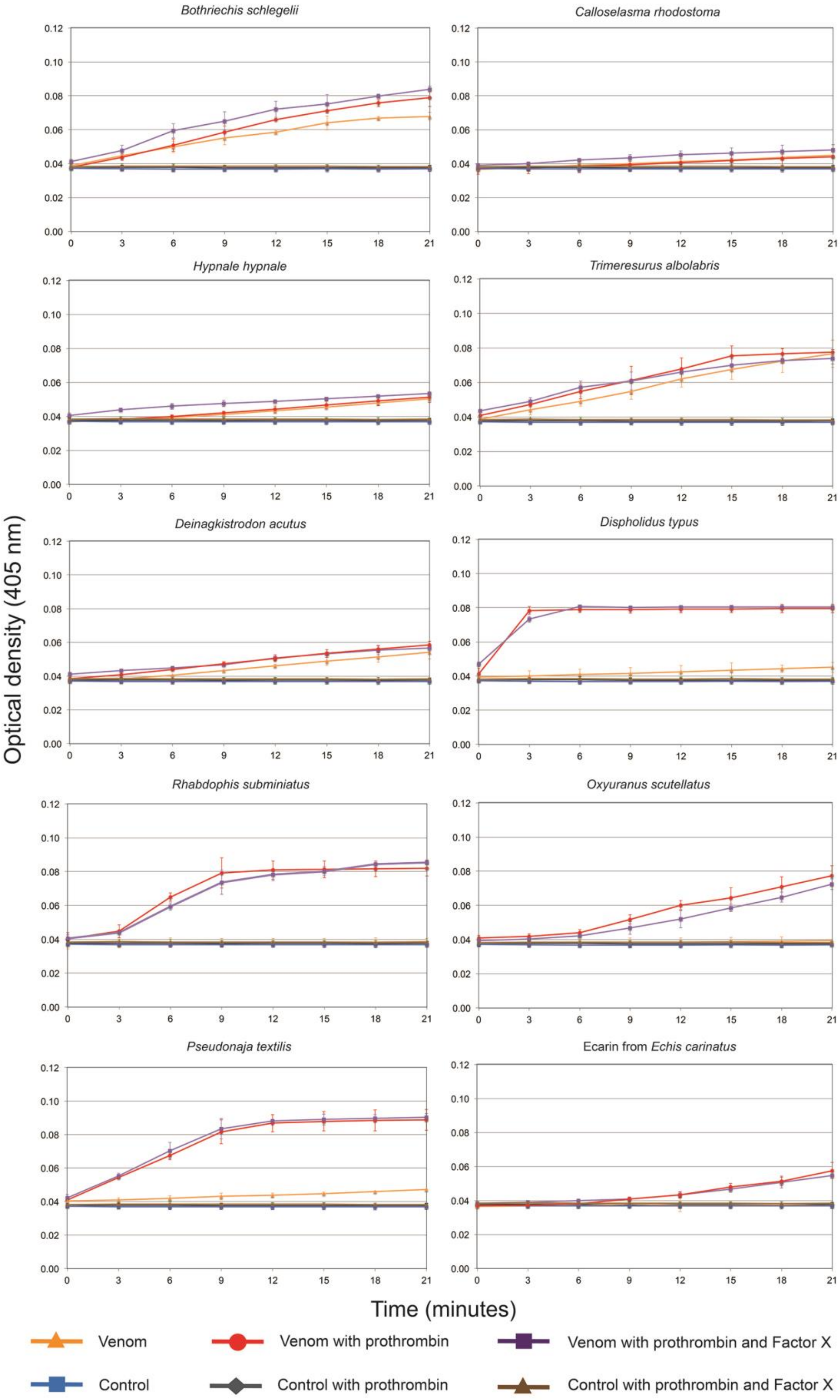

Supplementary Figure 8. Venom activity in the chromogenic assay in the presence of different clotting factors. Lines represent means of triplicate measurements and error bars represent SEMs. The lines displayed were used for the area under the curve analyses presented in Figure 2, following subtraction of appropriate control readings.

Supplementary Table 1. The species and localities of the snakes and the venoms used in this study.

Species	Family	Origin	Continent
Bitis gabonica	Viperidae (Viperinae)	West Africa (unknown locale)	Africa
Bitis arietans	"	Nigeria	Africa
Bitis nasicornis	"	West Africa (unknown locale)	Africa
Echis ocellatus	"	Nigeria	Africa
Echis pyramidum leakeyi	"	Kenya	Africa
Echis coloratus	"	Egypt	Africa
Echis carinatus sochureki	"	UAE	Asia
Echis carinatus	"	India	Asia
Cerastes cerastes	"	Egypt	Africa
Proatheris superciliaris	"	Tanzania	Africa
Atheris ceratophora	"	Tanzania	Africa
Causus maculatus	"	Nigeria	Africa
Causus rhombeatus	"	South Africa	Africa
Vipera ammodytes	"	unknown locale	Europe
Vipera aspis	"	France	Europe
Trimeresurus albolabris	Viperidae (Crotalinae)	Thailand	Asia
Tropidolaemus wagleri	"	Thailand	Asia
Deinagkistrodon acutus	"	China	Asia
Calloselasma rhodostoma	"	captive bred (unknown origin)	Asia
Hypnale hypnale	"	Sri Lanka	Asia
Crotalus horridus	"	USA	N. America
Crotalus viridis	"	USA	N. America
Sistrurus catenatus	"	USA	N. America
Agkistrodon bilineatus	"	Mexico	N. America
Bothriechis schlegelii	"	Costa Rica	N. America
Bothrops asper	"	Costa Rica	N. America
Bothrops jararaca	"	Brazil	S. America
Lachesis muta	"	Brazil	S. America
Aspidelaps lubricus	Elapidae	captive bred (unknown origin)	Africa
Dendroaspis angusticeps	"	Tanzania	Africa
Dendroaspis viridis	"	West Africa (unknown locale)	Africa
Dendroaspis polylepis	"	captive bred (unknown origin)	Africa
Naja multifasciata	"	Cameroon	Africa
Naja nubiae	"	unknown locale	Africa
Naja nigricollis	"	Togo	Africa
Naja haje	"	Morocco	Africa
Naja annulifera	"	unknown locale	Africa
Naja mossambica	"	unknown locale	Africa

Naja melanoleuca	"	Malawi	Africa
Naja nivea	"	South Africa	Africa
Naja pallida	"	captive bred (unknown origin)	Africa
Naja atra	"	China	Asia
Naja kaouthia	"	Thailand	Asia
Ophiophagus hannah	"	Thailand	Asia
Bungarus caeruleus	"	Sri Lanka	Asia
Enhydrina schistosa	"	Malaysia	Asia
Hydrophis cyanocinctus	"	Malaysia	Asia
Micrurus nigrocinctus	"	Costa Rica	N. America
Pseudechis australis	"	Australia	Australasia
Pseudonaja textilis	"	Australia	Australasia
Oxyuranus scutellatus	"	Papua New Guinea	Australasia
Acanthophis antarcticus	"	Papua New Guinea	Australasia
Micropechis ikaheka	"	Papua New Guinea	Australasia
Laticauda colubrina	"	Papua New Guinea	Australasia
Boiga irregularis	Colubrinae	Papua New Guinea	Australasia
Dispholidus typus	"	South Africa	Africa
Rhabdophis subminiatus	Natricinae	Hong Kong	Asia

Supplementary Table 2. The procoagulant potency of various snake venoms to normal citrated human plasma and their capability to clot plasma deficient in Factor X or prothrombin.

Species	MCD-P dose in normal plasma (µg ± SD)	Clotting of factor-deficient plasma			
		Factor X		Prothrombin	
		1x MCD-P dose	10x MCD-P dose	1x MCD-P dose	10x MCD-P dose
Echis ocellatus	0.09 (± 0.01)	✔	✔	✗	✗
Echis carinatus	0.49 (± 0.02)	✔	✔	✗	✗
Echis carinatus sochureki	0.35 (± 0.02)	✔	✔	✗	✗
Echis pyramidum leakeyi	0.44 (± 0.03)	✔	✔	✗	✗
Echis coloratus	17.50 (± 1.36)	✔	✔	✗	✗
Crotalus horridus	8.53 (± 0.87)	✔	✔	✔	✔
Bothrops asper	0.07 (± 0.01)	✔	✔	✔	✔*
Bothrops jararaca	4.40 (± 0.54)	✔	✔	✔	✔
Lachesis muta	2.29 (± 0.31)	✔	✔	✔	✔
Bothriechis schlegelii	2.07 (± 0.22)	✔	✔	✔	✔*
Calloselasma rhodostoma	1.12 (± 0.09)	✔	✔	✔	✔
Hypnale hypnale	7.18 (± 0.91)	✔	✔	✔	✔
Trimeresurus albolabris	7.66 (± 0.99)	✔	✔	✔	✔
Deinagkistrodon acutus	2.54 (± 0.40)	✔	✔	✔	✔
Dispholidus typus	0.03 (± 0.01)	✔	✔	✗	✗
Rhabdophis subminiatus	2.38 (± 0.19)	✔	✔	✗	✗
Oxyuranus scutellatus	0.34 (± 0.02)	✔	✔	✗	✗
Pseudonaja textilis	0.03 (± 0.01)	✔	✔	✗	✗

MCD-P dose refers to the dose of venom required to clot 200 µl of normal human citrated plasma in 60 seconds without the addition of cofactors. ±SD indicates standard deviation of triplicate results. Green ticks indicate that the venom clotted Factor X- or prothrombin-deficient human citrated plasma at 1 or 10 times the MCD-P dose in 60 seconds, except where asterisks are displayed, which indicate that clotting was delayed and occurred between one and five minutes (*B. asper:* 154 seconds; *B. schlegelii:* 86 seconds). Red crosses indicate that the venom failed to clot the plasma after five minutes.

Supplementary Table 3. The procoagulant potency of various snake venoms to human plasma and their neutralisation by different antivenoms.

Species	MCD-P (µg)	Neutralising MCD dose (µl antivenom)			
		EchiTAbG	SAIMR boomslang	CSL polyvalent	anti-ecarin
Echis ocellatus	0.09	✔ **10**	✔ 10	✗ NE	✔ 30
Echis carinatus	0.49	✔ 10	✔ 30	✗ NE	✔ 10
Echis carinatus sochureki	0.35	✔ 10	✔ 10	✗ NE	✔ 10
Echis pyramidum leakeyi	0.44	✔ 10	✔ 10	✗ NE	✔ 10
Echis coloratus	17.50	✔ 10	✔ 10	✗ NE	✗ NE
Crotalus horridus	8.53	✔ 10	✔ 30	✗ NE	✗ NE
Bothrops asper	0.07	✔ 10	✔ 30	✗ NE	✗ NE
Bothrops jararaca	4.40	✔ 10	✔ 30	✗ NE	✗ NE
Lachesis muta	2.29	✔ 30	✗ NE	✗ NE	✗ NE
Bothriechis schlegelii	2.07	✔ 30	✔ 30	✗ NE	✗ NE
Calloselasma rhodostoma	1.12	✗ NE	✗ NE	✗ NE	✗ NE
Hypnale hypnale	7.18	✗ NE	✗ NE	✗ NE	✗ NE
Trimeresurus albolabris	7.66	✔ 30	✔ 30	✗ NE	✗ NE
Deinagkistrodon acutus	2.54	✔□□ 30	✗ NE	✗ NE	✗ NE
Dispholidus typus	0.03	✔ 1	✔ **0.1**	✗ NE	✔ 1
Rhabdophis subminiatus	2.38	✔ 10	✔ 30	✗ NE	✗ NE
Oxyuranus scutellatus	0.34	✗ NE	✗ NE	✔ **0.1**	✗ NE
Pseudonaja textilis	0.03	✗ NE	✗ NE	✔ **0.1**	✗ NE

MCD-P dose refers to the dose of venom required to clot 200 µl plasma in 60 seconds (see Supplementary Table 2). Green ticks indicate that the antivenom prevented clot formation in the MCD-P assay at the dose displayed. Red crosses and NE indicate not effective. Doses tested for all antibodies were 0.1, 1, 10 and 30 µl. EchiTAbG – monospecific anti-*Echis ocellatus* antivenom (25 mg/ml); SAIMR boomslang – monospecific anti-*Dispholidus typus* antivenom (75 mg/ml); CSL polyvalent – polyspecific anti-Australian elapid (including *Oxyuranus scutellatus* and *Pseudonaja textilis*) antivenom (87.5 mg/ml); anti-ecarin – antibodies generated by immunisation with the *Echis*-specific SVMP prothrombin activator ecarin (25 mg/ml). Homologous combinations, i.e. *Echis ocellatus* vs *Echis ocellatus* antivenom, are highlighted by yellow shading. Control antibodies (IgG [25 mg/ml] purified from normal horse, sheep and rabbit serum) failed to prevent clotting by any venom at the highest dose tested (30 µl).
